# The CIN-TCP transcription factors promote commitment to differentiation in Arabidopsis leaf pavement cells via both auxin-dependent and independent pathways

**DOI:** 10.1371/journal.pgen.1007988

**Published:** 2019-02-11

**Authors:** Krishna Reddy Challa, Monalisha Rath, Utpal Nath

**Affiliations:** Department of Microbiology and Cell Biology, Indian Institute of Science, Bangalore, India; Peking University, CHINA

## Abstract

Cells in organ primordia undergo active proliferation at an early stage to generate sufficient number, before exiting proliferation and entering differentiation. However, how the actively proliferating cells are developmentally reprogrammed to acquire differentiation potential during organ maturation is unclear. Here, we induced a microRNA-resistant form of TCP4 at various developmental stages of Arabidopsis leaf primordium that lacked the activity of TCP4 and its homologues and followed its effect on growth kinematics. By combining this with spatio-temporal gene expression analysis, we show that TCP4 commits leaf cells within the transition zone to exit proliferation and enter differentiation. A 24-hour pulse of TCP4 activity was sufficient to impart irreversible differentiation competence to the actively dividing cells. A combination of biochemical and genetic analyses revealed that TCP4 imparts differentiation competence by promoting auxin response as well as by directly activating *HAT2*, a HD-ZIP II transcription factor-encoding gene that also acts downstream to auxin response. Our study offers a molecular link between the two major organ maturation factors, CIN-like TCPs and HD-ZIP II transcription factors and explains how TCP activity restricts the cell number and final size in a leaf.

## Author summary

Cells in a young organ primordium proliferate to generate sufficient number, before they exit division cycle and enter differentiation programme at later stages. While factors that drive cell cycle progression have been identified and studied in detail in diverse eukaryotic species, developmental factors that promote exit from division and entry into differentiation are less known, especially in the plant kingdom. Here, we show that the class II TCP proteins, notably TCP4, irreversibly reprogram the mitotic cells to exit division and acquire differentiation competence by auxin response as well as direct activation of *HAT2* transcription. Our work offers a molecular link between class II *TCP* and *HD-ZIP* II genes during the cell differentiation and leaf maturation.

## Introduction

Cells in an early organ primordium undergo active proliferation to generate sufficient number, while increasingly more cells exit division cycle and enter differentiation at later stages of organ growth, thus ensuring a mature organ with defined size [1,2]. While genes that drive cell cycle progression have been identified and studied in detail, developmental regulatory factors that promote the exit from cell division and entry to differentiation during organ morphogenesis are less known. Arabidopsis leaf epidermis acts as an ideal system to study this process due to its easy accessibility and since proliferation and differentiation take place in different regions of the same primordium during its development [3–5]. Moreover, the absence of cell migration and cell death as contributory factors to leaf epidermal morphogenesis makes the leaf surface, and the constituent cells, easily traceable during blade expansion [6,7].

Early leaf growth is sustained by cell division and mitotic cell expansion, collectively referred to as proliferation, which takes place throughout the primordium. As the primordium increases in size, the domain of this proliferation zone increases initially and is then maintained at constant size [4]. As cell number increases within the proliferation zone, the distal cells lose division potential and form a transition zone where they acquire differentiation competence [8]. Once the transition to differentiation is acquired, the pavement cells, which are the primary constituent cells of the lamina, do not revert back to division cycle under normal circumstances and start expanding in size to form the distal-most expansion zone. As more cells exit the transition zone, the relative size of the expansion zone increases and that of the proliferation zone decreases, giving the transition zone an appearance of a moving front in the basipetal direction [4,9]. All these three growth zones have diffused boundaries that overlap with the adjacent zones.

Cell number in a mature leaf depends on the size of the proliferation zone, the rate of proliferation within it and the rate at which cells exit proliferation and enter differentiation within the transition zone; perturbation in any of these parameters leads to altered cell number and final leaf size [10,11]. Many transcription factors have been identified over the years that promote cell proliferation and leaf size, the important ones being AINTEGUMENTA (ANT), GROWTH-REGULATING FACTORS (GRF) and GRF-INTERACTING FACTORS (GIF) [10,12,13]. Their inactivation results in smaller leaves with fewer cells while their over-expression yields larger leaves due to longer/ faster proliferation. The proteins that suppress cell proliferation in a leaf primordium have also been identified and include DA1/DAR, BIG BROTHER and the class II TCP (TEOSINTE BRANCHED 1, CYCLOIDEA, PROLIFERATING CELL FACTOR1/2) transcription factors [14–18].

The *TCP* genes are predicted to encode non-canonical, basic helix-loop-helix transcription factors [19,20] that regulate multiple aspects of plant development [21]. Five miR319-regutated class II TCP proteins in Arabidopsis, namely TCP2, 3, 4, 10 and 24, and their orthologues in snapdragon and tomato, regulate leaf morphogenesis by limiting the number of pavement cells [8,14,15]. In the Arabidopsis *jaw-D* plants where the level of these five *TCP* transcripts are reduced due to the overexpression of endogenous miR319a, leaf size is increased as a result of excess number of pavement cells, whereas leaves with elevated level or activity of these TCP proteins are smaller in size with reduced cell number due to precocious differentiation [9,17,22,23]. Based on these functional studies, together with the localization of their transcripts in the transition zone [14,15], it has been hypothesized that these class II TCP proteins promote differentiation potential in the proliferating leaf cells within their expression domain [24]. However, so far this has not been tested experimentally, primarily due to the high degree of functional redundancy among these proteins.

TCP-mediated cell differentiation and organ maturation has been linked to the biosynthesis and response pathways of the major phytohormone, auxin [21]. Auxin controls cell proliferation and maturation in a context-dependent manner. Plants expressing a dominant, repressive form of TCP15 display up-regulation of multiple auxin biosynthesis genes *YUCCA1*, *4* and *6*, suggesting that TCP15 is a negative regulator of auxin biosynthesis [25]. Similarly, TCP3 inhibits auxin response by inducing *IAA3* (*INDOLE-3-ACETIC ACID INDUCIBLE*3)*/ SHY2* (*SHORT HYPOCOTYL2*), a negative regulator of auxin signaling, and *PIN1* (*PIN-FORMED1*), *5*, *6*, *AUX1* (*AUXIN RESISTANT1*), the polar auxin transporters [21,26]. By contrast, TCP4 induces auxin response by activating *YUC5*, an auxin biosynthetic gene [27]. Even though the regulation of auxin response by TCP proteins has been studied in some detail, the role of auxin in cell maturation and transition zone formation has been studied only in roots [1] but not in leaves.

The role of the plant-specific homeodomain-leucine zipper (HD-ZIP) transcription factors has been implicated in leaf morphogenesis. The HD-ZIP members are classified into four sub-groups (I to IV) based on sequence similarity within the DNA-binding homeodomain [28,29]. Function of the class III HD-ZIP proteins such as REVOLUTA (REV), PHABOLUSA (PHB), PHAVOLUTA (PHV), ARABIDOPSIS THALIANA HOMEOBOX 8 (ATHB8) and ATHB15 is the most well-studied among the family members and their role has been linked to the establishment of leaf/ embryo polarity and meristem maintenance [30–32]. Members of the class II subgroup including HOMEODOMAIN ARABIDOPSIS THALIANA1 (HAT1) and HAT2 have been implicated in growth adaptation to the environmental signals and in organ maturation in response to hormone signaling [33–35]. Recent reports show that the class II HD-ZIP proteins ATHB4 and HAT3 physically interact with REV and form a functional repression complex which suppresses the miR165/166 expression, thereby establishing the adaxial/abaxial polarity in leaves [36]. Interestingly, ATHB4 and HAT3 are downstream targets of REV, pointing to a complex interconnection among the *HD-ZIP* genes during leaf morphogenesis. In addition, some members of the class II *HD-ZIP* genes, specifically *HAT1/2*, are activated by auxin response and promote cell expansion and organ maturation [33,34,37,38]. Plants over-expressing either *HAT1* or *HAT2* produce smaller leaves with reduced cell number and elongated hypocotyl cells [34,37,38], which is also seen in plants with gain-of-function of class II *TCP* genes [22,23,27]. However, it is not clear whether and how the HD-ZIP II proteins are linked to other cell differentiation factors in promoting organ maturation.

Here, we induced a dominant form of TCP4 protein at various developmental stages of Arabidopsis leaf primordium that lacked class II TCP function and followed its effect on leaf growth kinematics. By combining this with spatio-temporal expression of endogenous TCP4, we show that the class II TCP proteins irreversibly reprogram the mitotic cells to exit division and acquire differentiation competence within the transition zone. Induction of cell maturation is mediated by auxin response as well as by direct activation of *HAT2* transcription by TCP4.

## Results

### Altered number and size of pavement cells in the leaves with modified TCP activity

**Fig 1:**
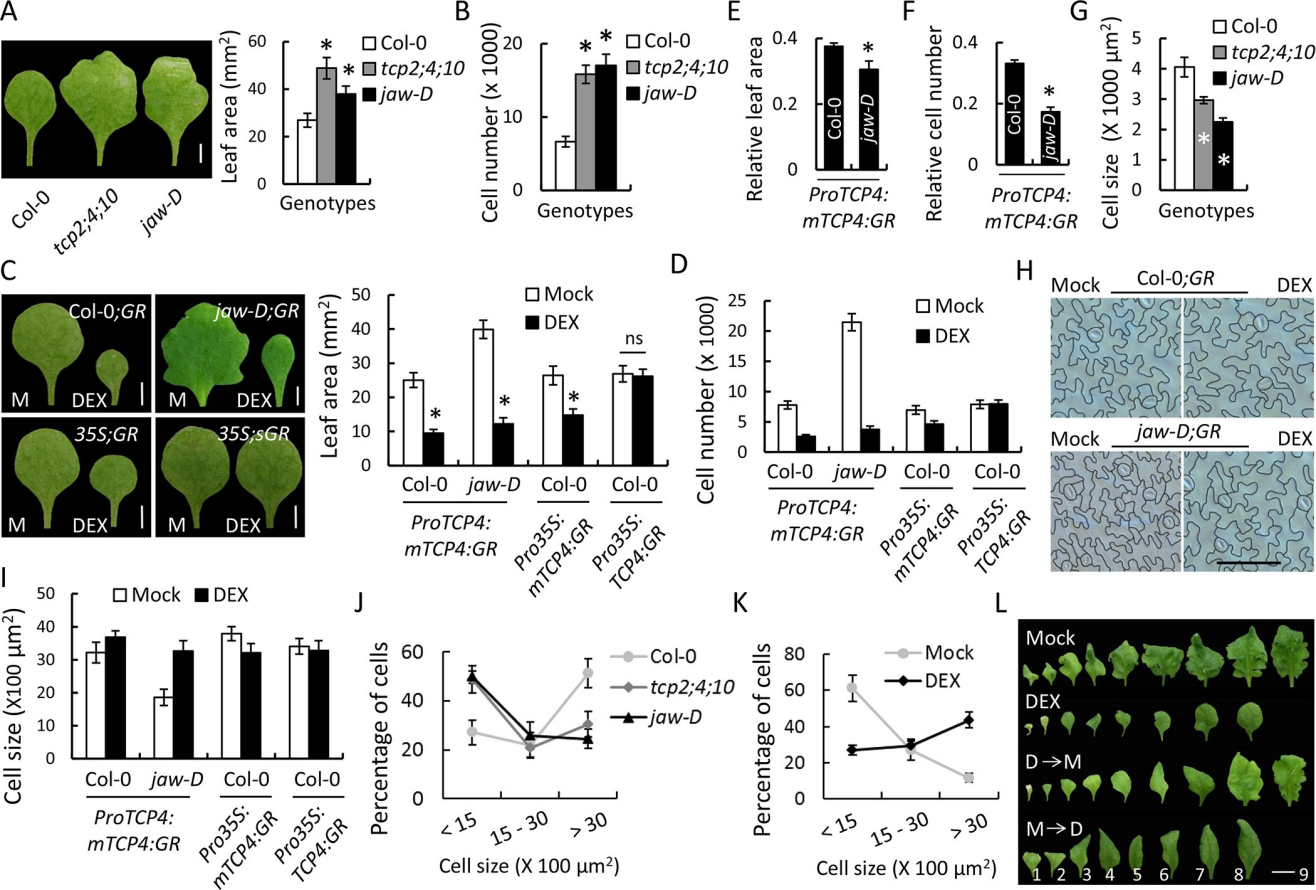
Perturbation in number and size of pavement cells in leaves with altered activity of miR319-targeted *TCP* genes. (A) and (B) Mature first leaves (A, left), average area of their lamina (A, right), and number (B) of their abaxial pavement cells. Sample size (N), 10–12. Scale bar, 2 mm. (C) and (D) Images of mature first leaves (C, left) from plants grown in the absence (M, Mock) or presence (DEX) of 12 μM dexamethasone and average area of their lamina (C, right), and number (D) of their abaxial pavement cells. N, 12–15. Scale bar, 2 mm. Col-0;*GR*, *jaw-D*;*GR*, *35S;GR* and *35S;sGR* indicate Col-0;*ProTCP4*:*mTCP4*:*GR*; *jaw-D*;*ProTCP4*:*mTCP4*:*GR*, Col-0;*Pro35S*:*mTCP4*:*GR* and Col-0;*Pro35S*:*TCP4*:*GR*, respectively. (E) and (F) Mature leaf area (E) and number of pavement cells (F) from DEX-treated Col-0;*ProTCP4*:*mTCP4*:*GR* and *jaw-D*;*ProTCP4*:*mTCP4*:*GR* plants relative to respective mock-treated values. Note that the relative value for *jaw-D*;*ProTCP4*:*mTCP4*:*GR* is smaller than that of Col-0;*ProTCP4*:*mTCP4*:*GR* since the denominator (mock value) for the former is more than that for the latter. (G) Average pavement cell area of leaves shown in (A). (H) and (I) Outline of the abaxial pavement cells (H) of leaves shown in (C) and their average area (I). Scale bar, 100 μm. For (G) and (I), total 125–150 cells per leaf at different regions were measured and averages from 5–8 leaves are shown. (J) and (K) Frequency distribution of pavement cell size in mature first leaves based on their size, of indicated genotypes (J), and of *jaw-D*;*ProTCP4*:*mTCP4*:*GR* plants grown without (Mock) or with (DEX) 12 μM dexamethasone (K). (L) Mature rosette leaves from 32-day old *jaw-D*;*ProTCP4*:*mTCP4*:*GR* plants grown in mock control (Mock), in continuous 12 μM dexamethasone (DEX), in dexamethasone for first 10 days and then transferred to mock (D→M), and in mock for first 10 days and then transferred to dexamethasone (M→D). The numbers indicate leaf positions. Scale bar, 1 cm. Error bars, * and ns indicate SD, p <0.05 and not significant, respectively. Unpaired Student’s *t*-test was used to determine significant differences.

The class II *TCP* genes are heterochronic regulators of leaf development and their function has been implicated in the repression of cell division [22] and the promotion of differentiation [17]. In order to examine the specific role of the five redundant, miR319-targeted, class II *TCP* genes–*TCP2*, *3*, *4*, *10* & *24*—in cell division/ differentiation of Arabidopsis leaves, we compared the mature first leaf pair and their epidermal cells of wild type (Col-0) with those in the loss-of-function mutants *tcp2;4;10* [27] and *jaw-D*, where all the five *TCP* transcripts are down-regulated due to the over-expression of miR319a [15]. The *tcp2;4;10* and *jaw-D* leaves measured >1.5 times larger than the Col-0 leaves (Fig 1A), as was reported previously [9,17]. The number of the abaxial epidermal cells in the mutant leaves increased ~2.5-fold (Fig 1B), suggesting that the increased mutant leaf area is due to an excess of cell proliferation. To determine if class II TCP activity is sufficient to repress proliferation, we induced a miR319-resistant, dexamethasone (DEX)-inducible form of TCP4 protein (mTCP4-GR) expressed under *ProTCP4* and *Pro35S* [27]. DEX-grown, homozygous *ProTCP4*:*mTCP4*:*GR* or *Pro35S*:*mTCP4*:*GR* plants in the Col-0 background (hereafter referred to as Col-0;*GR* or *35S;GR*, respectively) showed smaller leaves with reduced cell number (Fig 1C and 1D and S1A–S1D Fig). A more pronounced relative reduction in the final leaf size (by ~70%; Fig 1C and 1E) and cell number (by >80%; Fig 1D and 1F) were observed in the DEX-grown *ProTCP4*:*mTCP4*:*GR* plants in the *jaw-D* background (hereafter referred to as *jaw-D;GR*) compared to the mock treatment, demonstrating that TCP4 is a strong cell proliferation inhibitor. Similar negative effect of TCP4 on leaf size and cell number was also observed in three independent transgenic lines where three different gain-of-function forms of TCP4 were expressed constitutively under a leaf primordia-specific promoter (*ProBLS*:*rTCP4*:*GFP*) [17], a ubiquitous promoter (*Pro35S*:*TCP4*:*3F6H*) [39] or the endogenous promoter (*ProTCP4*:*TCP4*:*VP16*) [23] (S1F–S1I Fig).

Concomitant with the increased number, the area of mature pavement cells reduced to almost half in *jaw-D* and *tcp2;4;10* leaves (Fig 1G and S2A Fig). Induction of TCP4 activity, however, had little effect on the final cell area in the Col-0;*GR* and *35S;GR* leaves (Fig 1H and 1I and S1E Fig) and merely restored the cell size defect found in the *jaw-D;GR* leaves to the wild-type level (Fig 1I and S2B Fig). Pavement cell area also remained unaltered in the three other dominant lines of TCP4, *ProBLS*:*rTCP4*:*GFP*, *Pro35S*:*TCP4*:*3F6H* and *ProTCP4*:*TCP4*:*VP16* (S1J and S1K Fig). These results suggest that the class II TCP proteins are required for pavement cell maturation during leaf development but are not sufficient to promote pavement cell expansion *per se*, even though they do so in the hypocotyl [27]. Contrary to the miR319-resistant inducible form of TCP4, constitutive expression of a miR319-susceptible form of TCP4 protein in the homozygous *Pro35S*:*TCP4*:*GR* plants (hereafter referred to as *35S;sGR*) showed no effects on leaf size, cell number and pavement cell area (Fig 1C, 1D and 1I), demonstrating that the effects of mTCP4:GR induction described above resulted specifically from miR319 activity on the class II *TCP* transcripts and not from artifacts due to GR fusion or to the site of insertion [27].

The requirement of *TCP* genes for developmental cell maturation was also apparent in the reduced differentiation status of the pavement cells in the *TCP* loss-of-function leaves. The abaxial surface of mature Col-0 leaf is composed mostly of large epidermal cells with jigsaw shape (Fig 1H, S2A and S2C Fig), a characteristic marker for differentiation [17,40]. By contrast, mature *tcp2;4;10* and *jaw-D* leaves were composed mostly of smaller cells (Fig 1J, S2A and S2C Fig), a differentiation defect that was totally rescued by TCP4 induction (Fig 1K). However, induction of TCP4 in the Col-0 background did not alter the cell size distribution (S2D Fig).

### Kinematic analysis of TCP-mediated leaf growth

**Fig 2:**
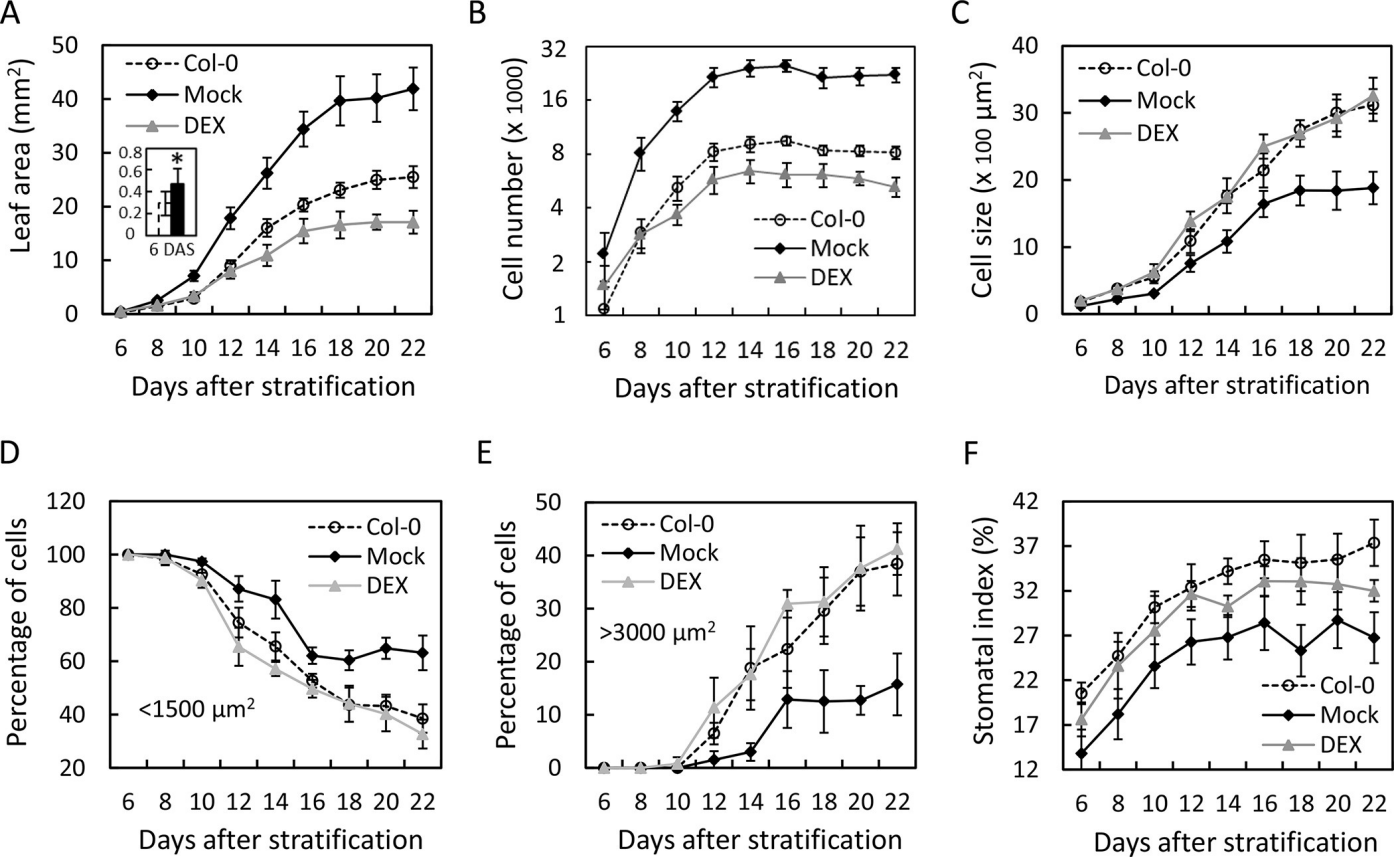
Kinematic growth analysis of leaves with altered TCP4 activity. (A) to (F) Kinematic growth parameters of the first leaf pair of *jaw-D*;*ProTCP4*:*mTCP4*:*GR* plants grown in the absence (Mock) or presence (DEX) of 12 μM dexamethasone are compared to the wild type (Col-0) control. (A) Average leaf area. Magnified data at 6 days after stratification (DAS) for Col-0 (open bar) and mock-grown *jaw-D;GR* (filled bar) leaves are shown in inset. (B) Average number of abaxial pavement cells per leaf (note that the Y-axis scale is in log_2_ scale). (C) Average area of abaxial pavement cells. (D) and (E) Frequency distribution of smaller (D, <1500 μm^2^) and larger (E, >3000 μm^2^) pavement cells are shown. For each time point, total 30–40 cells per leaf at base, mid or tip [5,41] were measured and averages from 5–7 leaves are shown. (F) Stomatal index expressed as percentage of total epidermal cells. N, 9–12 leaves. Error bars indicate SD and * indicate p <0.05.

The results described above suggest that the class II TCP proteins restrict cell number and promote cell maturation during leaf development. To examine their role in cell proliferation/ maturation at early growth phase, we compared the kinematics of growth of the first pair of *jaw-D;GR* leaves under TCP4 inductive and non-inductive conditions. Col-0 leaf blade expanded slowly at an early growth stage, from 6–10 days after stratification (DAS), during which the average area of the abaxial pavement cells remained small (~500 μm^2^ or less) and their number increased exponentially (Fig 2A–2C, S3A and S3B Fig). At 10 DAS, the blade acquired ~10% of its final area and ~75% of its final pavement cell number. Later, the blade expanded at an exponential rate up to 16–18 DAS, before reaching a plateau at ~22 DAS (Fig 2A and S3A Fig). The pavement cells also expanded faster during this period, and continued to do so beyond 22 DAS (Fig 2C and S3B Fig). The number of pavement cells reached its maximum at the mid-log phase of blade expansion and no further proliferation was observed beyond 12 DAS (Fig 2B), at which stage the blade acquired approximately half of its final area. These kinematics results are in general agreement with the earlier reports on Arabidopsis leaf published by other laboratories [5,41].

The overall trend of the growth kinematics of *jaw-D* leaf blade was by and large similar to Col-0, with a slow (6–10 DAS), exponential (10–18 DAS) and saturating (18–22 DAS) phase. However, the *jaw-D* blades started expanding at a rate faster than that of Col-0 from the earliest stage measured (6 DAS; inset in Fig 2A), and consequently its area remained higher throughout the growth. The increased *jaw-D* blade area was due to excess number, and despite reduced size, of the pavement cells. The *jaw-D* cells accumulated at a faster rate than Col-0, but the proliferation ceased at almost similar growth stage (~12 DAS) in both the genotypes, resulting in ~2.5 times more cells in the mature *jaw-D* leaf compared to Col-0 (Figs 1B and 2B). As in Col-0, the size of the *jaw-D* pavement cells remained small in the slow phase (6–10 DAS) and then increased exponentially until 16 DAS, before saturating rather abruptly (Fig 2C). However, the average area of *jaw-D* pavement cells remained 1.6 ± 0.16 fold smaller than that in Col-0 throughout the growth phase of the leaf (S1 Table).

All the cellular defects of the *jaw-D* leaf were rescued by continuous induction of TCP4 throughout the growth phases (Fig 2A–2C and S3A Fig). The DEX-grown *jaw-D;GR* blade expanded at the same rate as Col-0 until 12 DAS and then at a slower rate, before ceasing to expand at 18 DAS to a final area that was smaller than the Col-0 blade (Fig 2A and S3A Fig). Upon TCP4 induction, the pavement cells accumulated in the *jaw-D;GR* blade for almost the same duration (till ~12 DAS) as in Col-0, but at a slower rate, resulting in fewer cells at maturity compared to Col-0 (Figs 2B and 1D). While both blade area and cell number were smaller than the Col-0 values in the *jaw-D;GR* leaves grown under DEX, the average size of the pavement cells was rescued up to the Col-0 value at all stages of leaf growth (Figs 2C and 1I).

The growth kinematics described above shows that class II TCP activity is essential for reducing the total number, and for increasing the average size, of the pavements cells during leaf development. A growing leaf blade, however, is composed of cells of heterogeneous size and proliferation status; the smaller, dividing cells are located at the proximal end and the larger, differentiating cells towards more distal side [4]. To examine if TCP4 activity is specific to a stage of cell maturation, we determined the proportions of smaller (arbitrarily defined as <1500 μm^2^ in area) and larger (>3000 μm^2^) pavement cells in expanding Col-0 lamina (S2C Fig) and compared with *jaw-D*. During early growth (6–8 DAS), nearly all Col-0 pavement cells measured <1500 μm^2^, and their proportion sharply declined to ~30% at 22 DAS as the blade expanded (Fig 2D and S3C Fig). The larger cells started appearing at 10 DAS and steadily increased to ~40% at 22 DAS (Fig 2E). The fraction of the smaller cells in the *jaw-D;GR* blade without TCP4 induction, however, declined at a slower rate and stabilized at >60% at 16 DAS (Fig 2D and S3D Fig). Consequently, the *jaw-D;GR* larger cells increased in proportion rather slowly and reached the maximum value of little over 10% at 16 DAS (Fig 2E). TCP4 induction in the *jaw-D;GR* blade restored the proportions of smaller and larger cells close to the Col-0 levels at all growth stages (Fig 2D and 2E and S3E Fig).

Taken together, these kinematic results suggest that a major function of the class II TCP proteins is to convert the smaller pavement cells to larger cells, possibly by promoting their proliferation to differentiation competence. The differentiation-inducing activity of the TCP proteins is also apparent in the altered stomatal index (the proportion of epidermal cells assuming stomatal lineage), a measure of leaf maturation [41], in the mutant leaves. 20.5 ± 1.2% Col-0 epidermal cells assumed stomatal identity at 6 DAS, a value that steadily increased during leaf maturation and reached to a maximum value of 37.4 ± 2.6% at 22 DAS (Fig 2F). However, only 13.8 ± 2.7% *jaw-D;GR* cells were converted to stomatal lineage at 6 DAS, and this value increased to a maximum of 26.7 ± 2.8% at 22 DAS. Induction of TCP4 activity in the *jaw-D;GR* leaves nearly restored the defect in stomatal index to wild-type level.

### Temporal control of leaf growth by TCP4

All *jaw-D;GR* rosette leaves were smaller with smoother margin when grown with continuous presence of DEX (Fig 1L), indicating that TCP4 induction is active throughout the vegetative growth. However, when *jaw-D;GR* seedlings were grown under inductive condition for first 10 days and then transferred to non-inductive medium, the first few leaves resembled those grown under continuous DEX, but the leaves that emerged later showed progressively more *jaw-D*-like phenotype. Conversely, when the seedlings were grown under non-inductive condition for first 10 days and then transferred to inductive condition, the first few leaves resembled *jaw-D* leaves but the later leaves were smaller with smoother margin (Fig 1L). This suggests that DEX application can be used to induce TCP4 activity at any point of time during plant development.

**Fig 3:**
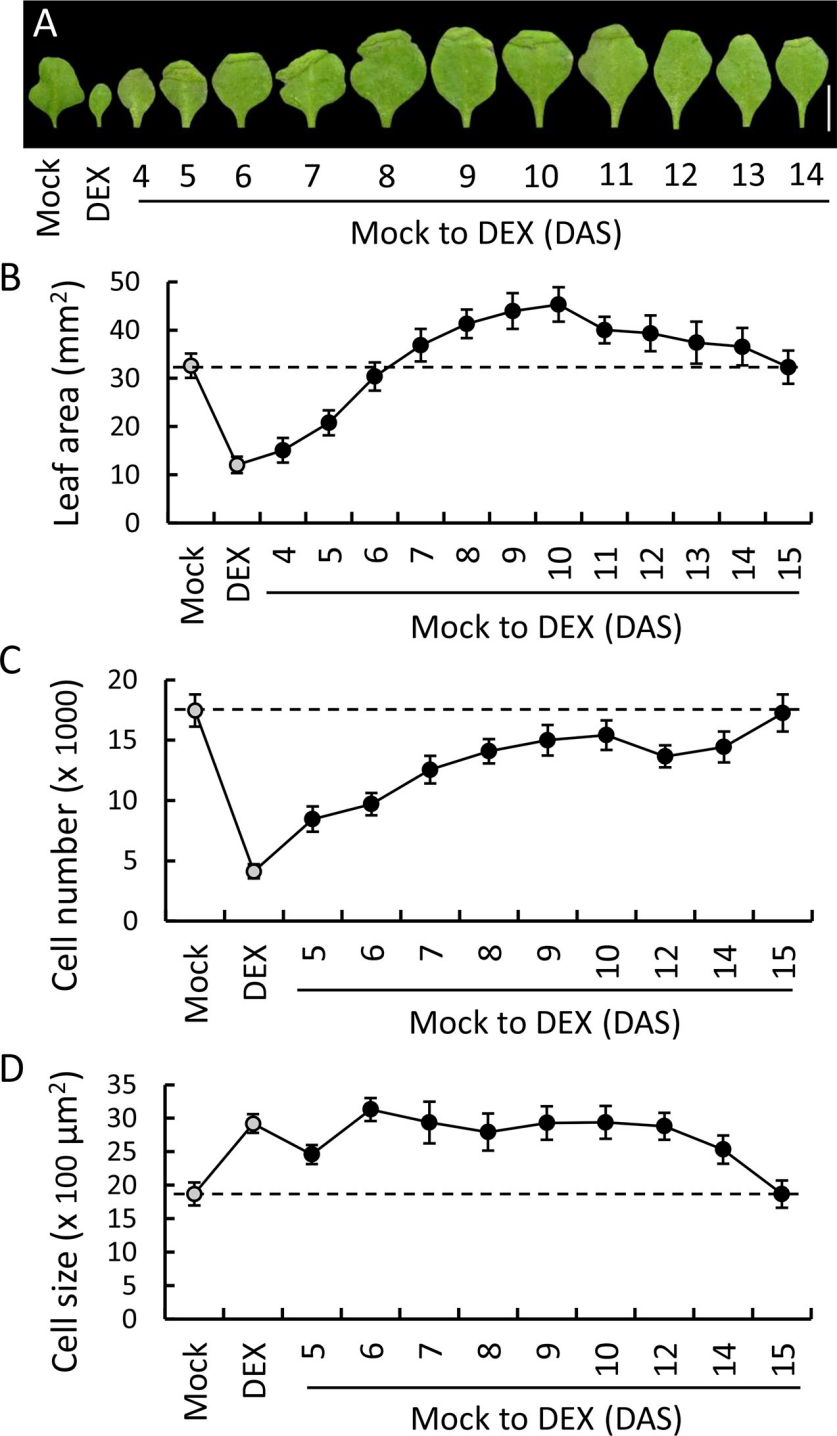
Temporal induction of cell differentiation by TCP4. TCP4 activity was induced at indicated DAS in the *jaw-D;ProTCP4*:*mTCP4*:*GR* seedlings by shifting them from mock to dexamethasone (12 μM)-containing medium and the mature first leaf at 29 DAS was analyzed. (A) Images of mature first leaves after shifting them from mock to DEX at indicated DAS. Leaves grown in continuous absence (Mock) or presence (DEX) of dexamethasone are shown as controls. (B) to (D) Average leaf area (B), and average number (C) and size (D) of abaxial pavement cells of leaves that are shown in (A). N, 12–15. For (D), total 120–140 cells per leaf were measured and averages from 5–7 leaves are shown. Mock and DEX (gray filled circles) indicate values for plants continuously treated with either mock solvent or dexamethasone, respectively. Broken lines are drawn through the values of the mock-treated controls. Error bars indicate SD. Scale bar, 5 mm.

To determine the developmental timing of TCP4 function during leaf maturation, we induced TCP4 activity in the *jaw-D;GR* leaves after growing them in the non-inductive medium for various number of days after stratification (4 to 15 DAS; Fig 3 and S4A Fig) and compared their blade area, cell number and cell size at maturity (29 DAS). Without TCP4 induction, the *jaw-D;GR* lamina grew to 32.6 ± 2.5 mm^2^ that contained 17450 ± 1336 pavement cells of average area 1870 ± 170 μm^2^ (Fig 3). On the other hand, the lamina under continuous TCP4 induction grew to 12.0 ± 1.7 mm^2^ with much fewer cells (4130 ± 579) of the average area 2920 ± 140 μm^2^. The blade area progressively increased from 12.0 ± 1.7 mm^2^ to the non-inductive value of 32.6 ± 2.5 mm^2^ when TCP4 was induced at 4, 5 or 6 DAS, even though their final cell numbers were much fewer than the non-inductive value. When TCP4 was induced at 6 DAS, its blade area at maturity was close to the *jaw-D* value (30.4 ± 2.3 mm^2^), even though its cell number was almost half of that in *jaw-D* leaf (Fig 3A–3C). This is because the defect in the pavement cell size was rescued in these leaves to the wild-type value (3130 ± 170 μm^2^) (Fig 3D).

When TCP4 was induced at 7, 8, 9 or 10 DAS, the final *jaw-D;GR* blade area progressively increased to a maximum of 46 mm^2^, nearly 1.5-fold bigger than the *jaw-D* blade (Fig 3A and 3B and S5A and S5B Fig). When TCP4 was induced at 10 DAS, the mature first leaf had pavement cell number (15420 ± 1220) that was close to the *jaw-D* value, but the cells (2940 ± 240 μm^2^) were larger than the *jaw-D* cells (1870 ± 170 μm^2^), ultimately yielding a mature leaf that was bigger than even the *jaw-D* leaf. It may be noted here that the *jaw-D* leaves were at the peak of its exponential phase of proliferation at 10 DAS, when almost all pavement cells were smaller in size (<1500 mm^2^; Fig 2B and 2D). When TCP4 was induced at 11, 12, 13, 14 and 15 DAS, the mature blade area progressively reduced to the *jaw-D* value (Fig 3A and 3B). At 15 DAS of TCP4 induction, the blade area, cell number and cell area were comparable to the respective *jaw-D* values, demonstrating that TCP4 induction at 15 DAS or later failed to rescue the size defect of the *jaw-D* pavement cells. At 15 DAS, the *jaw-D* cells ceased to proliferate and the proportion of the smaller cells are at its minimum level (Fig 2B and 2D).

When TCP4 was induced at various developmental stages of Col-0*;GR* and *35S;GR* leaves, similar effect on final blade area was observed (S5C–S5E Fig). Leaves of both genotypes grew to ~26 mm^2^ under non-inductive condition and to 9.2 ± 2.0 mm^2^ (Col-0*;GR*) and 12.7 ± 1.0 mm^2^ (*35S;GR*) upon continuous TCP4 induction (Fig 1C and S5C–S5E Fig). When TCP4 was induced at 4, 6, 8, 9 and 10 DAS in these plants, the final blade area progressively increased to the un-induced value but, unlike in *jaw-D;GR*, never surpassed it. This is consistent with the observation that TCP4 failed to promote pavement cell expansion beyond the wild type level (Figs 1I and 3D). On the contrary to the Col-0*;GR* and *35S;GR* leaves, when DEX-induction was performed in the expanding *35S;sGR* blades, the final blade area remained more or less unaffected at all stages of TCP4 induction (S5C and S5F Fig), implying that the collective effects of TCP4 induction on *jaw-D;GR*, Col-0*;GR* and *35S;GR* leaf growth dynamics described above resulted from miR319 activity on the *TCP4* transcript.

These results, together with those shown in Fig 2, strongly suggest that TCP4 promotes maturation in proliferating pavement cells, perhaps by committing them to differentiation, within the developmental window of the proliferative phase.

### TCP4 irreversibly changes the cell fate from proliferation to differentiation

**Fig 4:**
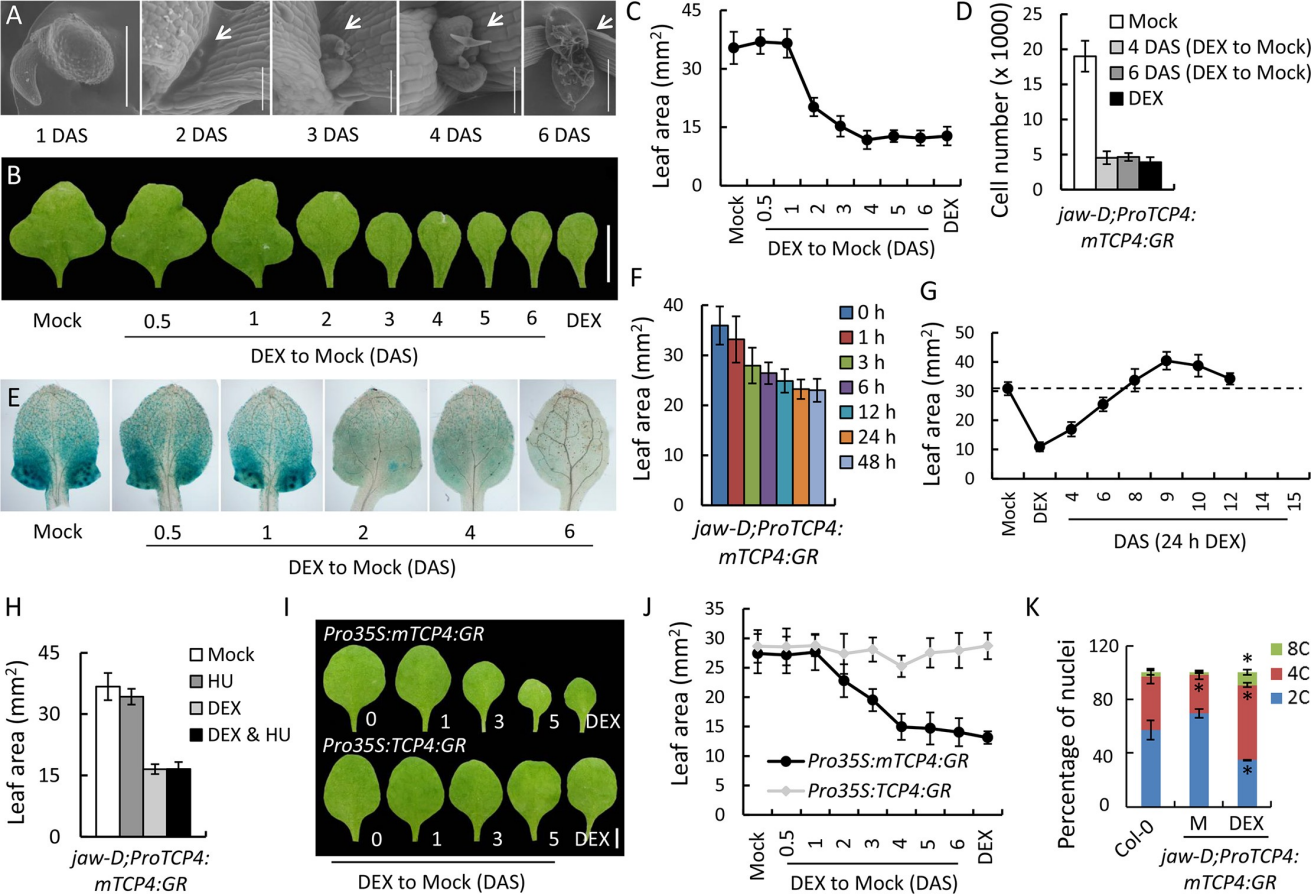
TCP4 irreversibly commits the proliferating cells of leaf primordia to differentiation. (A) Scanning electron micrographs of *jaw-D;ProTCP4*:*mTCP4*:*GR* seedlings after indicated DAS. A germinated seed (1 DAS) and the meristematic region of seedlings are shown. Scale bars, 500 μm (1, 6 DAS) and 100 μm (2, 3, 4 DAS). (B) Mature first leaf (29 DAS) of *jaw-D;ProTCP4*:*mTCP4*:*GR* seedlings grown in the presence of 12 μM dexamethasone for the indicated DAS and then shifted to medium without dexamethasone. Leaves grown in continuous absence (Mock) or presence (DEX) of dexamethasone are shown as controls. Scale bar, 5 mm. (C) Average size of leaves shown in (B). N, 12–15 leaves. (D) Number of pavement cells in leaves shown in (B). (E) GUS reporter analysis of first leaf from 8-day old *jaw-D;ProTCP4*:*mTCP4*:*GR* X *ProCyclinD3;2*:*GUS* seedlings grown in the presence of dexamethasone for the indicated DAS and then shifted to medium that lacks dexamethasone. Mock indicates seedlings grown without dexamethasone. (F) Average area of mature first leaves (N, 12–15) of *jaw-D;ProTCP4*:*mTCP4*:*GR* seedlings treated with 12 μM dexamethasone for indicated durations at 4 DAS. (G) Average area (N = 10–15) of mature first leaves of *jaw-D;ProTCP4*:*mTCP4*:*GR* seedlings treated with 12 μM dexamethasone for 24 hours at indicated DAS. Mock indicates no treatment; DEX, continuous dexamethasone treatment. (H) Average area of mature first leaves from *jaw-D;ProTCP4*:*mTCP4*:*GR* seedlings treated with 2 mM hydroxyurea (HU) and/or 12 μM dexamethasone (DEX) for 36 hours at 3 DAS. Mock indicates no treatment. N, 8–12 leaves. (I) and (J) Mature first leaf (I) from seedlings grown in the presence of dexamethasone for the indicated DAS and then shifted to medium that lacks dexamethasone, and their average size (J). N, 10–15 leaves. (K) Nuclear ploidy analysis of first leaf pair from 8-day old seedlings grown in the absence (Col-0, M) or presence (DEX) of 12 μM dexamethasone. Averages from biological triplicates are shown. Error bars indicate SD. * indicates p <0.05. Unpaired Student’s *t*-test was used.

**Fig 5:**
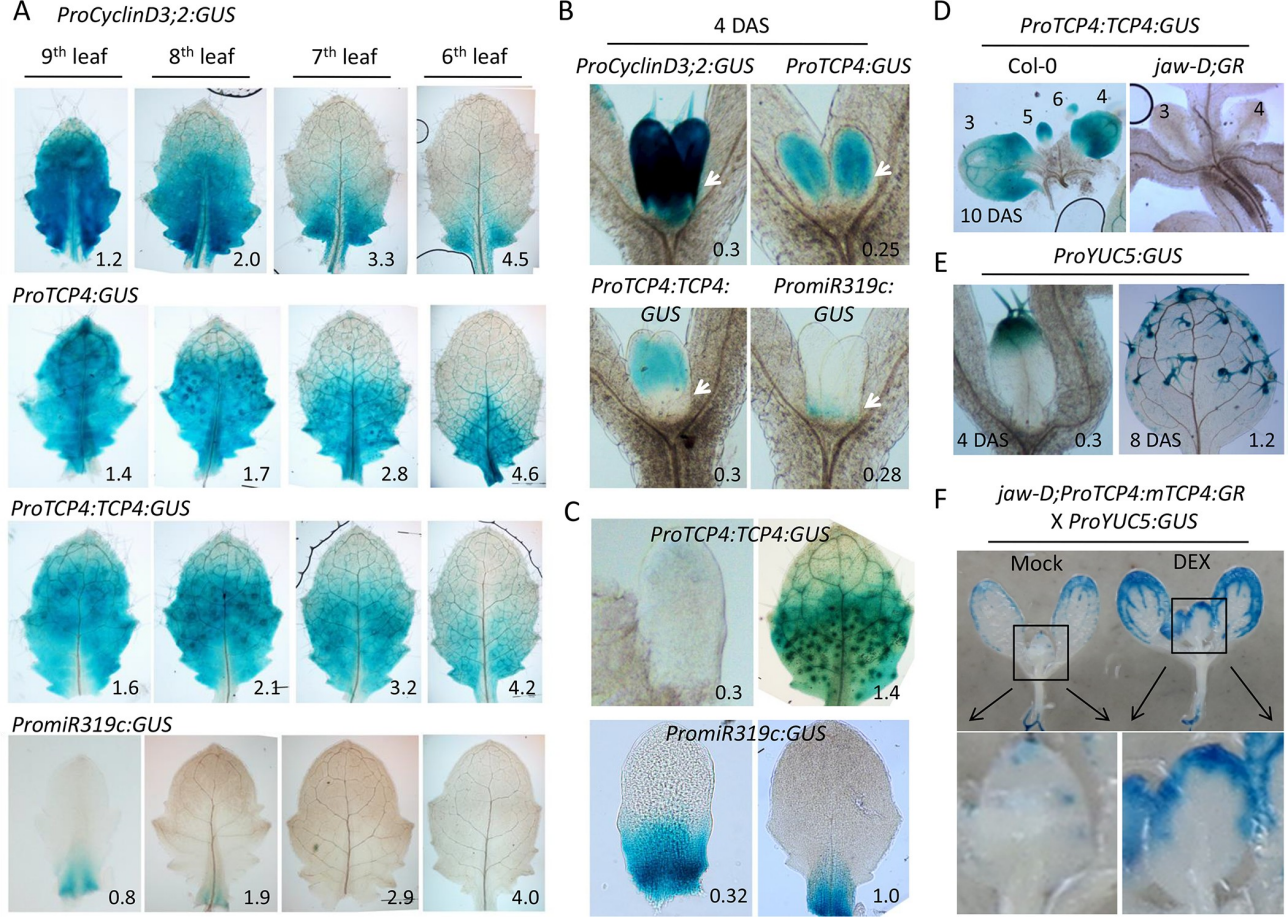
Spatio-temporal expression analysis of miR319-*TCP4* module. (A) and (B) GUS reporter analysis in young leaves of 19-day old seedlings (A) and in the first leaf pair in 4-day old seedlings (B). White arrows point to the leaf base. (C) GUS activity in the fifth leaf at two developmental stages. (D) *ProTCP4*:*TCP4*:*GUS* activity in 10-day old mock grown Col-0 and *jaw-D;GR* seedlings. (E) and (F) GUS activity of *YUC5* promoter in the first leaf (E) and in 5-day old seedlings grown in the absence (Mock) or presence (DEX) of 12 μM dexamethasone (F). The boxed regions in the top panel of (F) are magnified below to highlight GUS activity in the first leaf. Numbers indicate leaf length in mm (A) (B) (C) and (E) or leaf position (D).

Developmental cell maturation is a unidirectional process where the fully expanded pavement cells do not revert back to proliferative fate under normal circumstances. The transcripts of the miR319-regulated class II *TCP* genes that induce leaf differentiation are abundant in young lamina and their level steadily declines as the lamina matures [42]. Therefore, it is possible that these *TCP* genes activate the proliferation→differentiation transition in the pavement cells in an irreversible manner. To test this, we grew *jaw-D;GR* seedlings under TCP4 inductive condition for 0.5, 1, 2, 3, 4, 5 or 6 DAS, shifted them to non-inductive condition until 29 DAS (S4B Fig) and monitored leaf area and cell number of their mature first leaves (Fig 4A–4D). Arabidopsis seeds germinated (emergence of radicle) at 1 DAS and the first leaf pair initiated at 2 DAS (Fig 4A). When the plants were grown for 0.5 or 1 DAS in the presence of DEX, there was no effect of TCP4 induction on the final leaf area (Fig 4B and 4C), possibly because the leaf primordia were barely formed at 1 DAS. When TCP4 was induced for 2 DAS, the leaf margin defect was totally rescued (Fig 4B) and leaf area reduced to almost half (Fig 4C). TCP4 induction for 4 DAS yielded mature leaf as small as what was observed with continuous DEX induction (Fig 4B and 4C), suggesting that miR319-mediated inhibition of class II *TCP* transcripts for the first 2 days post leaf initiation (that is, 4 DAS), when the average length of leaf primordium was 274 ± 35 μm (Figs 4A and 5B), is crucial for normal leaf growth.

Consistent with the reduction of blade area, TCP4 induction for 4 DAS also reduced the pavement cell number to 4550 ± 910, similar to the value obtained with continuous TCP4 induction (Fig 4D). Lower cell proliferation activity was also reflected in the reduction of the cell cycle marker *CyclinD3;2* [43] in young leaves. Up to 1 DAS of TCP4 induction did not reduce the GUS activity to a noticeable extent in 8-day old *jaw-D;ProTCP4*:*mTCP4*:*GR* X *ProCyclinD3;2*:*GUS* seedlings (Fig 4E). However, the GUS activity much reduced when TCP4 was induced for 2 or 4 DAS, and totally disappeared at 6 DAS of induction.

In order to determine the minimum duration of TCP4 activity that is sufficient to induce differentiation commitment in proliferating cells, we provided pulse of TCP4 function of various durations from 0 to 48 hours in 4-day old *jaw-D;GR* seedlings and compared their mature leaf area at 29 DAS. Leaves without TCP4 induction grew to a maximum area of 36 ± 3.8 mm^2^ (Fig 4F). A 3-hour pulse of TCP4 induction reduced the area to a noticeable extent, and the area continued to reduce with longer pulses, finally stabilizing to a minimum value (23.2 ± 2.0 mm^2^) with a 24-hour pulse. This suggests that TCP4 activity for the duration of 24 hours is sufficient to impart commitment to differentiation in a dividing cell. Consequently, a 24-hour DEX pulse at 4, 6, 8, 9, 10 and 12 DAS (S6 Fig) altered the mature leaf area of mock-grown *jaw-D;GR* seedlings to nearly the same extent (Fig 4G) as in continuous DEX-induction starting from the respective DAS (Fig 3B). When we treated 3-day old *jaw-D;GR* seedlings with hydroxyurea, a widely used cell cycle inhibitor that induces reversible G1/S arrest [44,45], only for a duration of 36 hours, the mature first leaf grew as big as the untreated control (Fig 4H), implying that reversible cell arrest for a short while does not adversely affect the overall cell proliferation in a growing leaf. However, similar treatment with DEX reduced the final leaf size to a great extent (Fig 4H), suggesting that TCP4 initiates irreversible cell cycle arrest in a proliferating leaf.

When TCP4 activity was induced in the *35S*:*GR* seedlings for 0.5, 1, 2, 3, 4, 5 or 6 DAS, the final leaf area progressively reduced with increased duration of induction (Fig 4I, 4J and S7 Fig), as observed for *jaw-D;GR* leaves (Fig 4B and 4C). However, when similar induction experiment was performed on *35S;sGR* seedlings, the final leaf area remained unaltered, suggesting that miR319 activity at early stage of leaf growth contributes to blade expansion.

Increased cell maturation upon TCP4 induction was also reflected in the elevated level of nuclear ploidy, a typical cell differentiation marker [46], in cells of DEX-induced leaves. Flow cytometry analysis of the nuclei isolated from 8-day old Col-0 leaves revealed a ploidy distribution of 57.5% 2C, 39.6% 4C and 2.9% 8C cells (Fig 4K). In the *jaw-D;GR* leaves grown in mock condition, the proportion of 2C nuclei increased modestly to 69.5% and that of 4C (28.7%) and 8C (1.7%) nuclei decreased, suggesting a less differentiation status of this cell population. Conversely, when TCP4 function was induced in these leaves, the fraction of 2C nuclei reduced to 34.8%, with a concomitant increase in the proportion of 4C (55.7%) and 8C (9.5%) nuclei. Together these results demonstrate that TCP4 makes the proliferative cells exit division cycle and enter into endoreplication.

### MiR319-mediated spatial regulation of class II *TCP* transcripts establishes the transition zone

To correlate the function of the miR319-*TCP* module with their expression dynamics in expanding leaf blade, we localized *miR319c* and *TCP4* promoter activity and TCP4 protein at various growth stages of transgenic leaves expressing GUS reporter. In a 19-day old *ProTCP4*:*GUS* seedling, strong GUS activity was detected throughout the blade of the 9^th^ rosette leaf (1.2 mm long), which was progressively restricted towards the proximal end in the more mature 8^th^, 7^th^ and 6^th^ leaves (Fig 5A). Since the maturation states of younger to older leaves in an Arabidopsis rosette recapitulate the ontogeny of a leaf on a specific node [17], it can be concluded that TCP4 promoter activity progressively gets restricted towards leaf base as the blade differentiates, before disappearing completely, as observed previously for *TCP4* and its orthologues [8,14,47]. GUS activity in the *ProCyclinD3;2*:*GUS* leaves [43] resembled that of *ProTCP4*:*GUS* leaves (Fig 5A), suggesting that *TCP4* promoter is active in the proliferative zone of the expanding leaf blade.

GUS reporter was detected in a small domain at the base of the young 9^th^ leaf (0.8 mm long) of *PromiR319c*:*GUS* seedlings (Fig 5A), and expression was reduced and restricted to more proximal region in the 8^th^ leaf (1.9 mm long); no detectable GUS activity was found in the 7^th^ and the 6^th^ leaves. These results are in agreement with the earlier reports [48], and suggests that *miR319c* promoter is active during a small temporal window at the earliest stage of leaf growth. Consequently, the TCP4:GUS reporter activity in young *ProTCP4*:*TCP4*:*GUS* leaf at the 9^th^ position was not detected in the corresponding basal region where miR319c promoter is active at this stage, suggesting that miR319 activity excludes *TCP4* transcript [15] and its protein product (Fig 5A) from its expression domain.

To compare the relative expression domains of miR319c, *TCP4* transcript and TCP4 protein in young leaf primordia where miR319 promoter is active, we studied the GUS expression pattern in 4-day old transgenic seedlings where the first leaf pair just initiated (Fig 4A). In ~300 μm long first leaf, *TCP4* and *CyclinD3;2* promoters were active throughout the leaf, whereas the miR319 promoter was detected in a small region at the very base (Fig 5B and S8 Fig). Activity of the TCP4:GUS fusion protein was, however, detected only in the distal half of young leaf primordia (Fig 5B and 5D and S8 Fig), and was excluded from the proximal region where miR319c promoter is active. Similar expression patterns have been reported for endogenous *TCP4* transcript in wild-type [15], and for a miR319-susceptible form of *ProTCP4*:*GUS* transcript in transgenic leaf primordia [47].

In young fifth leaf primordium (~300 μm long), barely detectable TCP4:GUS activity was found towards the distal extreme (Fig 5C), implying that miR319 activity is present in a broader basal domain in this leaf. In agreement with this, strong GUS activity was detected across the basal half of a *PromiR319c*:*GUS* leaf primordium of similar length (Fig 5C). At a later growth stage, *PromiR319c* activity was restricted more towards the base, where it reduced the TCP4:GUS signal. Since TCP4:GUS activity retracted from the distal end in these leaves owing to the lack of *ProTCP4* activity in differentiated cells, the TCP4:GUS protein was detected in a band-like domain in the middle of the leaf with diffused proximal and distal boundaries (Fig 5A, 5C and 5D), which has been proposed as the transition zone where proliferating cells acquire differentiation competence [3,4,8,24]. Ectopic expression of miR319 throughout the leaf blade completely abolished the TCP4:GUS activity in the *jaw-D;GR* X *ProTCP4*:*TCP4*:*GUS* seedlings (Fig 5D and S8 Fig), highlighting the importance of class II TCP activity within the transition zone in leaf maturation.

Cells proximal to the transition zone are expected to remain proliferative whereas the distal cells would differentiate. In agreement with this, the promoter activity of *YUCCA5* (*YUC5*), a known differentiation marker [27,49], was initiated at the distal end in a ~300 μm long first leaf of 4-day old *ProYUC5*:*GUS* seedlings, and the expression extended towards more proximal direction as the leaf matured (Fig 5E). The GUS signal reduced when the class II *TCP* genes were down-regulated in the *jaw-D;GR X ProYUC5*:*GUS* leaves (Fig 5F), and increased when gain-of-function TCP4 activity was induced by DEX application, suggesting that the distal expression of *YUC5* in young leaves is indeed mediated by TCP4 activity.

### TCP4-induced auxin response is required for pavement cell maturation

**Fig 6:**
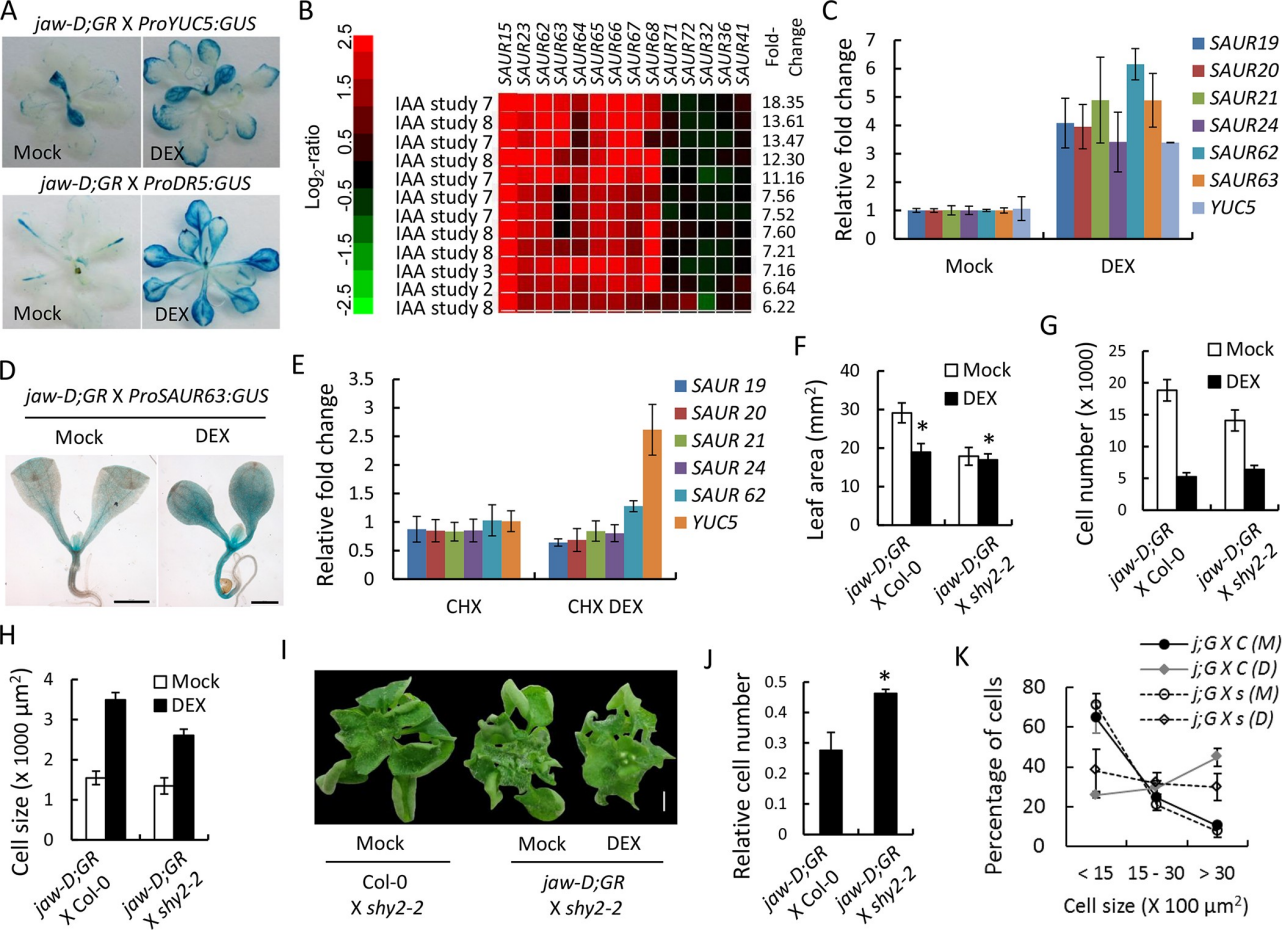
TCP4-mediated cell differentiation requires auxin signaling. (A) GUS reporter assay of 17-day old seedlings grown in the absence (Mock) or presence (DEX) of dexamethasone. (B) Expression analysis of 14 *SAUR* genes in previously reported microarray datasets of indole-3-acetic acid (IAA)-treated seedlings using the signature tool of Genevestigator database (*https*:*//www*.*genevestigator*.*com/gv/plant*.*jsp*). Top 12 transcriptome profiles of IAA-treated microarrays are shown. (C) Relative transcript levels of *SAUR* genes determined by RT-qPCR analysis in 9-day old *jaw-D;ProTCP4*:*mTCP4*:*GR* seedlings grown in the absence (Mock) or presence (DEX) of 12 μM dexamethasone. Averages from independent biological triplicates are shown. *YUC5* level has been used as a positive control. (D) GUS analysis of 4-day old seedlings grown on mock and DEX containing medium. Scale bar, 1 mm. (E) Relative transcript levels of indicated genes estimated by RT-qPCR analysis in 9-day old *jaw-D;ProTCP4*:*mTCP4*:*GR* seedlings treated either with 40 μM cyclohexamide (CHX) or with a combination of 40 μM cyclohexamide and 20 μM dexamethasone (CHX DEX) for 4 hrs. *PP2A/TUB2* was used as internal controls. (F) to (J) Average leaf area (F), number of total pavement cells (G), average pavement cell area (H) and relative cell number (J) in mature first leaves of indicated genotypes grown in the absence (Mock) or presence (DEX) of dexamethasone are shown. 30-day old rosettes pictures of indicated genotypes are shown in (I). Cell number in the DEX-grown leaf was normalized to that of Mock-grown leaf (J). (K) Frequency distribution of pavement cell size in mature first leaves. *j;G X C* and *j;G X s* indicate *jaw-D;ProTCP4*:*mTCP4*:*GR* X Col-0 and *jaw-D;ProTCP4*:*mTCP4*:*GR* X *shy2-2*, respectively. M and D indicate Mock and DEX, respectively. Total 130–150 cells from 5–7 leaves were analyzed for (H) and (K). Error bars indicate SD. Scale bar, 2 mm (I). N, 12–15 leaves (F).

The expression dynamics of TCP4 described above, together with the kinematic data (Figs 2–4), suggests that TCP4 activity imparts irreversible differentiation fate to the proliferating cells within the transition zone of an expanding leaf blade. Auxin is known to play a key role in tissue maturation by coordinating the cell division with differentiation [1,50]. TCP4 function has been linked to the auxin biosynthesis and response in hypocotyl cells [27]. We observed that TCP4 activation increased GUS reporter activity in both *jaw-D;GR X ProYUC5*:*GUS* and *jaw-D;GR X ProDR5*:*GUS* rosettes (Figs 5F and 6A), demonstrating that TCP4 promotes *YUC5* transcription and auxin response in developing leaves. In agreement with this observation, the levels of many *SAUR* and *HD-ZIP II* transcripts, which are known targets of auxin signaling [34,51,52], were altered upon TCP4 induction (S9 Fig) when a previously reported *jaw-D;GR* microarray dataset was analyzed [27]. Fourteen *SAUR* transcripts were also elevated upon heightened auxin response or upon external application of auxin (Fig 6B) in the microarray datasets available in the GENEVESTIGATOR database [33,34,51–53]. The *SAUR* and *HD-ZIP II* genes are known to be involved in multiple developmental events including cell proliferation and differentiation [34,37,50,51], suggesting a link between TCP4 function and auxin responsive genes in cell differentiation. RT-qPCR analysis demonstrated that the transcripts of *SAUR19*, *20*, *21*, *24*, *62* and *63* increased in the *jaw-D;GR* seedlings grown under constitutive TCP4 activation (Fig 6C). In addition, GUS reporter activity in the *jaw-D;GR X ProSAUR63*:*GUS* seedlings increased upon dexamethasone treatment (Fig 6D), suggesting that TCP4 activates the *SAUR* genes *in planta*. *SAUR* activation was abolished in the *jaw-D;GR* seedlings when TCP4 was induced for 4 h in the presence of cycloheximide, a general protein synthesis inhibitor (Fig 6E) [54], suggesting that the promotion of *SAUR* by TCP4 is an indirect effect, possibly mediated by elevated auxin response.

To examine whether the elevated auxin response by TCP4 is capable of inducing leaf cell maturity, we studied the effect of TCP4 induction on pavement cell area/number in *short hypocotyl2-2* (*shy2-2*), where cells are insensitive to auxin response due to the sequestration of AUXIN RESPONSE FACTORs (ARF6 and 8) by a dominant, non-degradable form of AUX/INDOLE-3-ACETIC ACID3 (IAA3) inhibitor protein [55]. Mature first leaves of *jaw-D;GR* X Col-0 heterozygous seedlings grew to 29 ± 2.6 mm^2^, consisting of 18850 ± 1670 pavement cells with average area of 1550 ± 165 μm^2^ (Fig 6F, 6G and 6H). TCP4 induction in these leaves reduced the blade area to 19.0 ± 2.2 mm^2^ and cell number to 5240 ± 650, while increased the average cell area to 3490 ± 180 μm^2^. The average area of mature *jaw-D;GR X shy2-2* leaves (Fig 6I) under non-inductive condition was 18.0 ± 2.3 mm^2^, with the total cell number 14080 ± 1660 and average cell area 1350 ± 200 μm^2^ (Fig 6F, 6G and 6H), possibly indicating a TCP-independent role for auxin response in leaf blade expansion. TCP4 induction, however, failed to reduce the *jaw-D;GR X shy2-2* leaf area to any noticeable extent (Fig 6F), even though it increased the pavement cell area ~2-fold, as in *jaw-D;GR* X Col-0 control leaves (Fig 6H). While TCP4 induction reduced the total pavement cell number by ~75% in the *jaw-D;GR* X Col-0 leaves, it reduced the *jaw-D;GR X shy2-2* cell number by ~50% (Fig 6G and 6J), suggesting that at least a part of the TCP4 effect on the repression of cell number is mediated by IAA3-dependent suppression of auxin signaling.

The pavement cell population of the mature *jaw-D;GR X* Col-0 and *jaw-D;GR X shy2-2* leaves showed similar size distribution under un-induced condition (Fig 6K), with ~70% cells of smaller area (<1500 μm^2^) and <10% cells of larger area (>3000 μm^2^). Upon TCP4 induction, the fraction of the smaller cells decreased to ~25% and that of larger cells increased to ~50% in *jaw-D;GR X* Col-0, indicating a rescue of the cell size defect by TCP4 activity. The extent of rescue was, however, less (~40% smaller cells and ~30% larger cells) in the *jaw-D;GR X shy2-2* leaves, suggesting that TCP4 partly requires auxin response to induce cell maturity.

### TCP4 activates *HAT2* independent of auxin response

**Fig 7:**
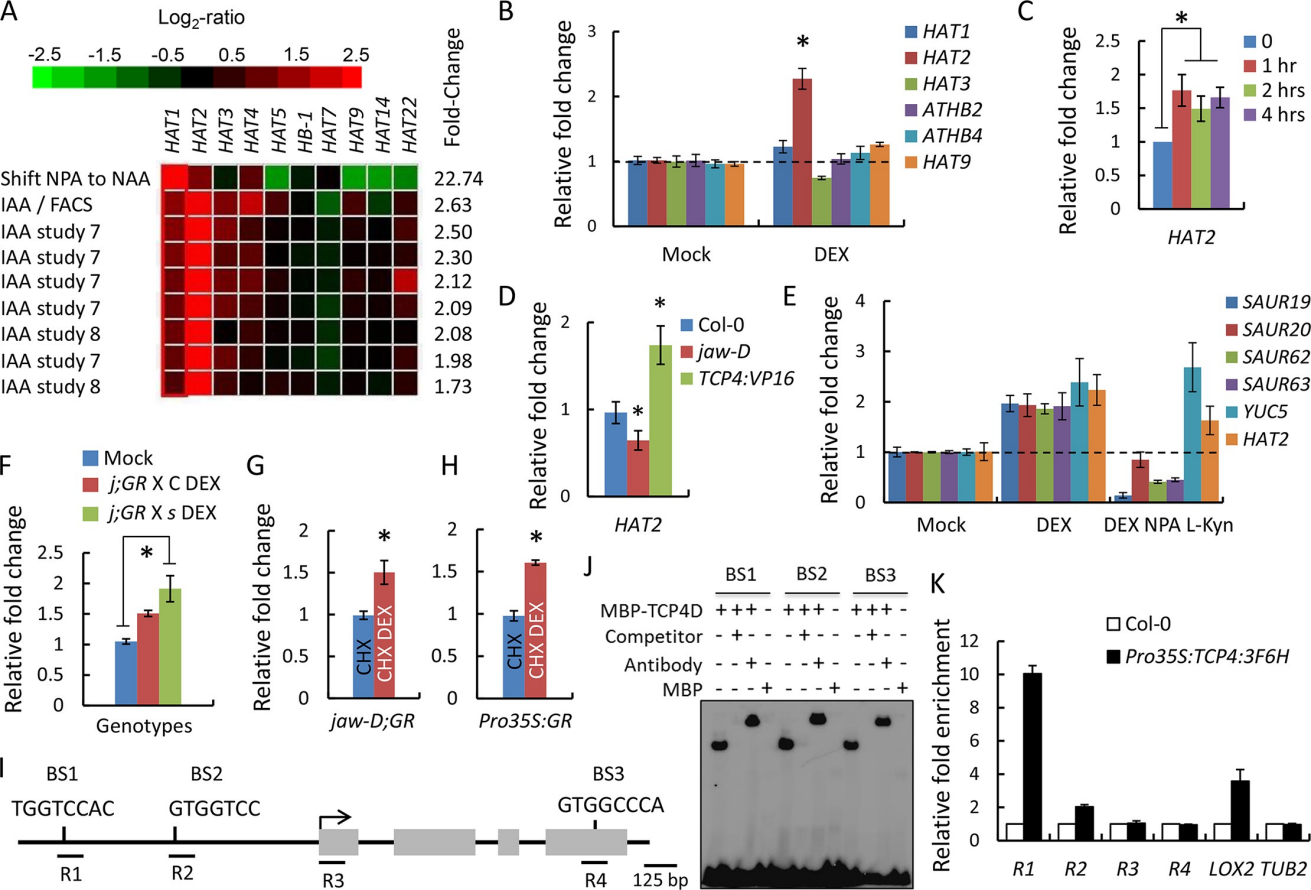
TCP4 directly activates *HAT2* transcription. (A) Comparative transcript levels of Class II *HD-ZIP* genes in Arabidopsis seedlings in previously reported microarray datasets upon auxin treatment using the signature tool of Genevestigator database (*https*:*//www*.*genevestigator*.*com/gv/plant*.*jsp*). Top 9 transcriptome profiles are shown. (B) and (D) Relative transcript levels of class II *HD-ZIP* genes in *jaw-D;ProTCP4*:*mTCP4*:*GR* (B) and *HAT2* in indicated genotypes (D) determined by RT-qPCR in 9-day old seedlings grown without dexamethasone (Mock) or in the continuous presence 12 μM dexamethasone (DEX). (C) RT-qPCR analysis of *HAT2* expression in 9-day old *jaw-D;ProTCP4*:*mTCP4*:*GR* seedlings treated with 12 μM dexamethasone for indicated durations. (E) RT-qPCR analysis of indicated transcripts in 9-day old *jaw-D;ProTCP4*:*mTCP4*:*GR* seedlings treated either with DEX alone or with DEX plus and combination of NPA and L-Kyn, pharmacological inhibitors of auxin transport and synthesis respectively, for 2 hrs. Solvent treatment (Mock) has been used a negative control. (F) *HAT2* transcript levels determined by RT-qPCR in 9-day old seedlings grown without (Mock) or with (DEX) 12 μM dexamethasone. *j;GR* X C indicate *jaw-D;ProTCP4*:*mTCP4*:*GR* X Col-0 and and *j;GR* X *s* indicate *jaw-D;ProTCP4*:*mTCP4*:*GR* X *shy2-2*. Transcript levels were initially normalized to *PP2A*/*TUB2* internal control and then compared to respective mock controls. (G) and (H) *HAT2* transcript levels analyzed by RT-qPCR in 9-day old *jaw-D;GR* (*jaw-D;ProTCP4*:*mTCP4*:*GR*) (G) or *Pro35S*:*GR* (*Pro35S*:*mTCP4*:*GR*) (H) seedlings treated either with 40 μM cyclohexamide (CHX) or with a combination of 40 μM cyclohexamide and 20 μM dexamethasone (CHX DEX) for 4 hrs. (I) Schematic representation of the *HAT2* locus. Exons are shown in gray box and translation start site is indicated with an arrow. BS1-BS3 represent the putative TCP4-binding motifs that are used for EMSA experiments in (J). R1-R4 indicate the regions that are used for ChIP-qPCR analysis shown in (K). (J) Electrophoretic mobility shift assay demonstrating the retardation of the oligonucleotides corresponding to the TCP4-binding sites in the *HAT2* locus shown in (I) by recombinant MBP-fused TCP4 domain (MBP-TCP4D). 250-fold of un-labeled oligonucleotides was used as competitors. For super shift, anti-MBP antibody was used. (K) ChIP-qPCR analysis of the *HAT2* chromatin regions R1-R4 (shown in I) with anti-FLAG antibody. *LOX2* and *TUB2* were used as positive and negative controls, respectively. For RT-qPCR analyses in (B-H) and Chip-qPCR in (K), averages of biological triplicates are shown. Error bars indicate SD. * indicates p <0.05, ns indicates not significant, Unpaired Student’s *t*-test was used.

*HAT2* and its closest homologue *HAT1* have been recognized as immediate auxin responsive genes and control cell proliferation in leaves and cell elongation in hypocotyls, functions that overlap with miR319-targeted *TCP* genes [22,27,33,34]. *HAT1* and *HAT2* transcripts were consistently up-regulated in multiple independent microarray datasets performed upon external auxin application (Fig 7A) [34]. *HAT2* transcript was also up-regulated at both 2 and 4 h of TCP4 induction in an earlier microarray experiment (S9 Fig) [27]. RT-qPCR analysis showed that the transcript of *HAT2* alone, and not of *HAT1* or the clade members, was up-regulated upon constitutive TCP4 induction in the *Pro35S*:*mTCP4*:*GR* seedlings (Fig 7B). *HAT2* was induced within 1 h of TCP4 induction (Fig 7C), and the level was maintained for at least 4 h. In addition, *HAT2* level was elevated in *TCP4*:*VP16* seedlings, a gain-of-function line of TCP4 with enhanced transcriptional activity [23], while that was significantly reduced in the loss-of-function line *jaw-D* (Fig 7D). Together, these results suggest that *HAT2* is an early downstream target of TCP4.

Since class II TCP proteins induce auxin biosynthesis [27,56] and *HAT2* is an early auxin responsive gene, it would appear that *HAT2* activation is an indirect effect of TCP4 induction. However, we found that TCP4 induction activates *HAT2*, but not the *SAUR* genes, in the *jaw-D;ProTCP4*:*mTCP4*:*GR* seedlings even when both auxin biosynthesis and polar auxin transport were inhibited by administering a combination of L-kynurenine and N-1-naphthylphthalamic acid (Fig 7E) [57]. Moreover, TCP4 induction in the *jaw-D;GR* X *shy2-2* seedlings, where auxin response was genetically depleted by expressing a degradation-resistant form of IAA3 [55], also promoted *HAT2* expression (Fig 7F). These results suggest that TCP4 is capable of promoting *HAT2* expression independent of auxin response, and possibly by direct transcriptional activation.

### *HAT2* is a direct target of TCP4

Consistent with this hypothesis, TCP4 induction increased *HAT2* transcript level in *jaw-D;ProTCP4*:*mTCP4*:*GR* and *Pro35S*:*mTCP4*:*GR* seedlings even when new protein synthesis was blocked by applying cycloheximide (Fig 7G and 7H). This suggests that TCP4 directly activates *HAT2* possibly by binding to its genomic locus. Analysis of *HAT2* genomic sequence yielded three putative TCP4 binding elements (BS1-3); two in the promoter region and the third in the coding region (Fig 7I). Electrophoretic mobility shift assay showed that recombinant MBP (maltose binding protein)-tagged TCP4 protein [20] binds to oligonucleotides corresponding to these elements with sequence specificity (Fig 7J). To test whether the TCP4 protein is recruited to the *HAT2* chromatin *in planta*, we performed chromatin immuno-precipitation (ChIP) assay in the *Pro35S*:*TCP4*:*3F6H* transgenic line using anti-FLAG antibody [39]. TCP4 recruitment was observed in the *HAT2* upstream regulatory regions that corresponded to the TCP4 binding motifs (Fig 7K), but no recruitment was found in the *HAT2* coding regions. Consistent with this result, formaldehyde-assisted isolation of regulatory elements (FAIRE) experiment [58] suggested that the *HAT2* chromatin assumed more open conformation in the regions corresponding to the putative TCP4-binding sites and not in a distant region when TCP4 was activated in the *Pro35S*:*mTCP4*:*GR* seedlings by dexamethasone treatment (S10A Fig).

Even though we identified one putative TCP4 binding site in the *HAT1* upstream regulatory region and another in the coding region (S10B Fig), we suspected that *HAT1* is not a target of TCP4 since its transcript level remained unaltered upon TCP4 induction (Fig 7B). ChIP assay also suggested that TCP4 protein is not recruited at the *HAT1* locus (S10C Fig). Based on these results, and on the observation of a general overlap in the expression levels of *TCP4* and *HAT2* at various developmental stages (S11 Fig), we conclude that *HAT2*, and not *HAT1*, is a direct target of TCP4.

### CIN-TCPs require HD-ZIP II transcription factors for limiting cell number and leaf size

**Fig 8:**
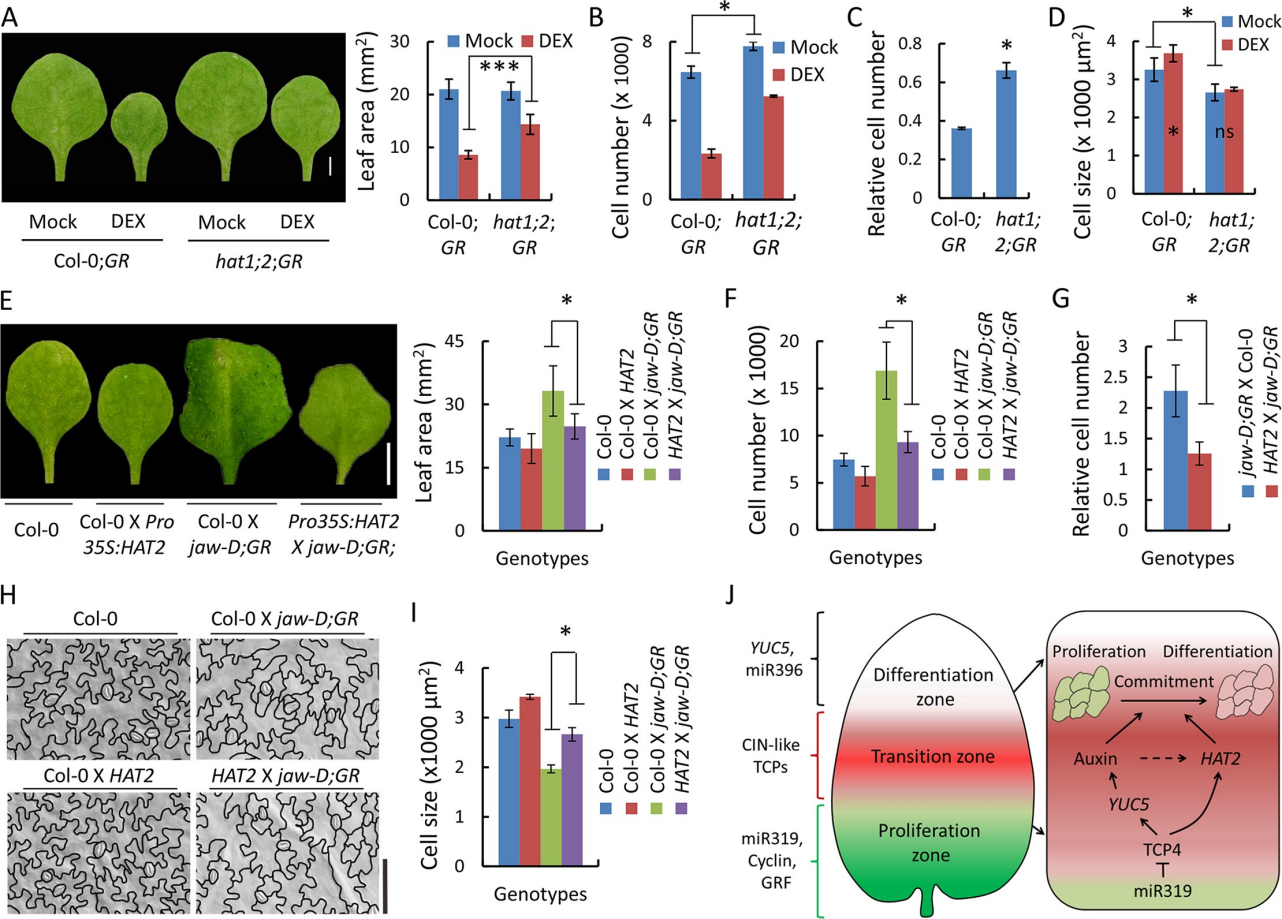
TCP4 requires the HD-ZIP II transcription factors for leaf maturation. (A) to (D) Mature first leaves (A, left) of plants grown without (Mock) or with (DEX) 12 μM dexamethasone, their average area (A, right), total number of pavement cells (B), cell number upon TCP4 induction relative to mock-induction (C) and average abaxial pavement cell area (D). Sample number, 10–13 leaves. Col-0;*GR* and *hat1;2;GR* indicate Col-0*;ProTCP4*:*mTCP4*:*GR* and *hat1;2;ProTCP4*:*mTCP4*:*GR*, respectively. For (A), *** indicates p <0.0001 (Unpaired Student’s *t*-test was performed). (E) to (I) Mature first leaves from 29-day old seedlings of indicated genotypes (E, left), their average area (E, right), total pavement cell number (F), cell number relative to Col-0 (G), outline of abaxial pavement cells (H) and their average area (I). N, 8–12 leaves. *jaw-D;GR* and *HAT2* indicate *jaw-D;ProTCP4*:*mTCP4*:*GR* and *Pro35S*:*HAT2*, respectively. For (D) and (I) total 125–150 cells per leaf were measured and averages from 3–7 leaves are shown. Error bars indicate SD. * indicates p <0.05. Unpaired Student’s *t*-test was used. (J) A schematic diagram proposed to describe the mechanism of TCP4-induced commitment to differentiation of proliferating cells in a wild type leaf. The proliferative basal zone (green) remains devoid of TCP4 activity due to the action of miR319 and expresses cyclins and *GRF* [22,65]. The transition zone (red) above the base expresses miR319-regulated class II TCP proteins that induce cell commitment within this region via auxin response as well as *HAT2* activation. TCP4 activity declines in the differentiated zone towards the tip (white), which expresses the direct targets of TCP4 such as *YUC5* and miR396 [22,27]. Scale bars, 2 mm (A), 5 mm (E) and 100 μm (H).

Both TCP4 and HAT2 restrict cell number in leaves and promote cell expansion in hypocotyl [27,34]. Since TCP4 directly activates *HAT2* (Fig 7), it is likely that TCP4 requires *HAT2* activity *in planta* to promote developmental cell maturation. To test this, we crossed the *jaw-D;GR* line to the *hat1;hat2* double mutant, since there is a high degree of functional redundancy between *HAT1* and *HAT2* and their single mutants cause little phenotypic alterations [59]. However, the dominant *jaw-D* phenotype was suppressed in the *jaw-D;GR* X *hat1;hat2* F_1_ individuals possibly due to transgene silencing, a phenomenon that commonly occurs with the viral 35S promoter [60,61]. To overcome this, we established the Col-0;*GR;hat1;hat2* line and analyzed its leaf parameters upon TCP4 induction. The Col-0;*GR* leaves, which resemble Col-0 under non-inductive condition [27], were reduced to ~38% in area upon TCP4 induction, primarily due to a corresponding reduction in the number of pavement cells (Fig 8A–8C) that were mildly but significantly larger than the un-induced cells (Fig 8D). Though the mature *hat1;hat2* leaves resembled wild type leaves in shape and size (Fig 8A), they were made up of an increased number of pavement cells that were smaller in area (Fig 8B and 8D), an indication of differentiation defect frequently observed in the mutants of *CIN-TCP* genes (Fig 1) [17]. When TCP4 was activated in the absence of *HAT1* and *HAT2* gene function in the *hat1;2;GR* line, leaf blade area reduced only to ~70% with a corresponding reduction in the pavement cell number. Further, TCP4 activation failed to sustain the increase in the pavement cell size in the absence of *HAT* function (Fig 8D). Taken together, these results suggest that TCP4 requires *HAT2*, and perhaps *HAT1* as well, to limit cell proliferation and leaf size.

It has been shown that overexpression of *HAT2* inhibits the leaf size and promotes the cell elongation in hypocotyl similar to that of TCP4 activity in leaf and hypocotyl, respectively [27,34,59]. *Pro35S*:*HAT2* line shows only a mild effect in reducing leaf size and cell number in the wild-type background (60). However, this line reduced the size of the *jaw-D* leaf by ~30% and its pavement cell number by ~50% (Fig 8E–8G). Further, *Pro35S*:*HAT2* rescued the small-size phenotype of the *jaw-D* pavement cells nearly to the wild type level (Fig 8H and 8I).

Since *HAT1* and *HAT2* are functionally redundant partners and their overexpression yields comparable changes in leaf and hypocotyl [33–35,37], we tested whether *HAT1* overexpression can also rescue *jaw-D* phenotype. Similar to TCP4 dominant effect (Fig 8A), overexpression of *HAT1* in the *Pro35S*:*JAIBA*:*GFP* line (*JAB*:*GFP*) [37] reduced leaf size to a third of the wild type value with ~60% decrease in pavement cell number (S12A–S12C Fig), demonstrating that *HAT1* is a strong cell proliferation inhibitor in developing leaves. Moreover, *HAT1* overexpression rescued the effect of class II *TCP* down-regulation on the leaf size and pavement cell number of the *jaw-D;GR* leaves nearly to the Col-0 values (S12A–S12D Fig). Intriguingly, overexpression of *HAT1* resulted in a significant decrease in pavement cell area (S12E and S12F Fig), a phenotype that contrasts TCP4 overexpression. This negative effect of *HAT1* on pavement cell size remained unaltered even in the *jaw-D;GR* background, possibly indicating an effect of *HAT1* independent of TCP4.

Taken together, these genetic interaction studies show that TCP4 induces pavement cell maturation in part via direct activation of *HAT2*, and not *HAT1*. However, *HAT1* (along with *HAT2*) is likely to be an indirect target of TCP4 as it is an auxin response gene (Fig 7A) [34] and TCP4 is known to promote auxin level through *YUC5* activation (Fig 6A) [27]. Consequently, overexpression of each of these clade-members (*HAT1* and *HAT2*) rescued *jaw-D* phenotype (Fig 8E–8I and S12 Fig).

## Discussion

Constitutive down-regulation of class II TCP activity resulted in the accumulation of more cells with smaller average area while increased TCP function reduced cell number without affecting the average size (Figs 1–3) [22], thus confounding the exact effect of TCP proteins on cell proliferation and expansion. Frequency distribution of the pavement cell population based on cell area identified increased proportion of smaller cells in the *jaw-D* and *tcp2;4;10* leaves, a defect that was rescued by TCP4 induction (Figs 1 and 2). Cell size in an organ primordium remains small as long as cells proliferate and starts to expand when they differentiate [2], suggesting that TCP4 converts the dividing cells to differentiation. This is further supported by our growth kinematics analysis that demonstrated that the effect of TCP4 on cell number is confined within the proliferation phase and TCP4 induction beyond this phase had no effects on final cell number and leaf area (Fig 3 and S5 Fig) [17], thus discounting any role for TCP4 in regulating differentiation-associated leaf cell expansion. TCP4 activity for the first two days of leaf initiation, when the primordium measured <300 μm in length, had the same effect on cell number and leaf size as observed with continuous TCP4 activity throughout the growth phase (Fig 4). Further, a 24-hour pulse of TCP4 activity in the leaf primordium reduced the final leaf area as much as the continuous TCP4 activity did (Fig 4). These results suggest that TCP4 imparts irreversible differentiation competence to the dividing cells of leaf primordium.

The mature miR319 is active at the base of young leaves (Fig 5A–5C) [48], restricting its target *TCP* transcripts towards more distal region of the blade [15,47]. Since the promoter activity of these *TCP* genes are excluded from the distal differentiated zone (Fig 5A) [62], their transcripts and protein products are restricted to a broad transition zone within a growing leaf blade that interfaces, and overlaps with, the more distal differentiation zone and the proximal proliferation zone, where they provide morphogenetic competence to the leaf blade (Fig 8J) [8,24].

To explain the precise role of class II TCP proteins in leaf cell proliferation/ maturation, here we propose a model (Fig 8J) that incorporates the expression domain of the miR319/*TCP* module and the function of the TCP proteins based on the growth kinematics results. The transcripts of class II *TCP* genes are undetectable at the base of the leaf primordia due to the activity of mature miR319, even though their promoters are active in this zone. Lack of TCP activity helps the basal cells maintain their mitotic status [4]. Distal to this proliferation zone, TCP proteins are active in the transition zone where they commit the dividing cells to exit division and acquire differentiation competence. As the basal region expands due to cell proliferation, more dividing cells are incorporated within the transition zone where they encounter TCP proteins that commit them to exit cell division cycle. Since TCP activity imparts irreversible differentiation status to the leaf cells in the transition zone (Figs 3 and 4), the cells in the distal differentiation zone, though devoid of TCP activity, remain committed to differentiation and start expanding.

Our model explains the leaf phenotype with reduced or elevated TCP activity. Due to the absence of TCP proteins from *jaw-D* leaves (Fig 5D), increased proportion of cells within the transition zone remain mitotic and therefore smaller in size (Fig 2D) since fewer cells exit the division cycle (Fig 2E). This yields a lower cell area when averaged over the entire lamina (Fig 2C). Higher proportion of mitotic cells in the *jaw-D* leaves ultimately results in increased cell number at maturity (Fig 2B). When TCP4 activity was induced in the transition zone of the *jaw-D;ProTCP4*:*mTCP4*:*GR* leaves, the proportion of cells exiting proliferation was restored to the wild-type level (Fig 2D and 2E), resulting in the correction of the defects in cell size and number. However, since *jaw-D;ProTCP4*:*mTCP4*:*GR* leaves express a dominant form of TCP4 that cannot be degraded by miR319 [15], the ectopic TCP4 activity at the base prematurely converts the proliferating cells to differentiated fate, thereby reducing the final cell number and leaf size at maturity (Fig 2).

Young leaf primordia with or without TCP4 induction contained nearly similar number of pavement cells (Fig 2B), but the TCP4-induced leaves had higher fraction of cells with increased ploidy compared to the un-induced leaves (Fig 4K). Increased nuclear ploidy resulting from endoreplication cycle is used as a differentiation marker since it commits the cells at G2 phase to bypass mitosis and directly enter into the S-phase [46]. The cyclin-dependent kinase inhibitors KRP1 and KRP2 inhibit entry to mitosis and promote the onset of endoreplication in leaves [63,64]. Further, *KRP1* is directly activated by TCP4 during leaf development [22], suggesting that KRP-induced endoreplication forms an important component of TCP-mediated onset of cell differentiation within the transition zone.

Suppression of the *GRF*s that redundantly promote cell proliferation in the leaf primordia is yet another likely mechanism of TCP-mediated restriction of cell number in leaves [12,65,66]. This possibility is further supported by a recent observation that the promoter of miR396, a microRNA that degrades the transcripts of several *GRF* genes, is activated by TCP4 [22]. However, the effects of the miR319-*TCP* module on cell proliferation and differentiation are distinct from those of the miR396-*GRF* module in several aspects. First, TCP activation restricts the pavement cell number without a noticeable increase in size, whereas lack of *GRF* products results in a reduction in pavement cell number with a concomitant increase in cell size. Second, lack of TCP activity increases the cell number as well as decreases its average size, whereas overexpression of GRF proteins increase cell number without affecting the size. Third, while the class II TCP proteins are expressed only within the transition zone, the activity of miR396 promoter in the leaf primordia is initiated in the distal expansion zone and that of its target proteins is detected in the entire leaf base including the proliferation zone [12,22,66]. These differences point to a GRF-independent function of TCP proteins in promoting cell differentiation. Nevertheless, a yet unidentified link between the activities of TCP and GRF proteins within the transition zone cannot be ruled out.

The GRF-independent TCP function is likely mediated by multiple parallel pathways that possibly converge to the commitment of proliferating cells to differentiation within the transition zone. One arm of this function is via auxin response originating from TCP-mediated *YUCCA* activation [27,56]. An auxin minimum has been shown to promote a cellular switch from division to differentiation in root transition zone [50], whereas auxin response appears to be required for TCP-mediated differentiation commitment (Figs 6 and 8J). Auxin response activates the transcription of *HAT2*, a gene that restricts cell number and leaf size in Arabidopsis [33,34,37,38]. Consistent with this, *hat1;hat2* leaves contain excess cells with reduced size (Fig 8B and 8D). Interestingly, the class II TCP proteins also promote division to differentiation switch in leaf pavement cells independent of auxin by directly activating *HAT2* transcription (Fig 7). These parallel pathways possibly ensure a robust *HAT* activation by TCP proteins. The downstream events of *HAT* activation triggering cell differentiation are currently unclear. The possibility of the HAT transcription factors directly promoting the KRP cell cycle inhibitors to initiate endoreduplication, as shown for TCP4 [22], needs to be tested.

## Materials and methods

### Plant materials and growth conditions

Col-0 was used as the wild type control in all experiments. The mutant lines *tcp2;4;10*, *jaw-D*, *shy2-2* and *hat1;2* were reported previously [9,15,33,55]. The Col-0;*Pro35S*:*mTCP4*:*GR #2* line was screened and established in the T_2_ generation. The cloning methodology and primers information was previously reported [27]. The homozygous transgenic lines Col-0;*ProTCP4*:*mTCP4*:*GR*, *jaw-D;ProTCP4*:*mTCP4*:*GR*, Col-0;*Pro35S*:*mTCP4*:*GR*, Col-0;*Pro35S*:*TCP4*:*GR*, *ProYUC5*:*GUS*, *ProDR5*:*GUS*, *ProCyclinD3;2*:*GUS*, *PromiR319c*:*GUS*, *ProSAUR63*:*GUS*, *Pro35S*:*JAB*:*GFP*, *Pro35S*:*HAT2*, *Pro35S*:*TCP4*:*3F6H*, *ProBLS*:*rTCP4*:*GFP* and *ProTCP4*:*TCP4*:*VP16* used in this study are all established lines reported previously [17,23,27,37,39,43,48,59,67,68]. The F_1_ genetic crosses of *jaw-D;ProTCP4*:*mTCP4*:*GR* X *ProYUC5*:*GUS*, Col-0 X *ProTCP4*:*GUS*, *jaw-D;ProTCP4*:*mTCP4*:*GR* X *ProTCP4*:*GUS*, Col-0 X *ProTCP4*:*TCP4*:*GUS*, *jaw-D;ProTCP4*:*mTCP4*:*GR* X *ProTCP4*:*TCP4*:*GUS*, *jaw-D;ProTCP4*:*mTCP4*:*GR* X *ProDR5*:*GUS*, *jaw-D;ProTCP4*:*mTCP4*:*GR* X *ProSAUR63*:*GUS*, Col-0 X *Pro35S*:*HAT2* and *jaw-D;ProTCP4*:*mTCP4*:*GR* X *Pro35S*:*HAT2* were used in this study. Col-0;*ProTCP4*:*mTCP4*:*GR* line was established in the *hat1;2* background by genetic cross in F_4_ generation. The homozygous *jaw-D;ProTCP4*:*mTCP4*:*GR*;*Pro35S*:*JAB*:*GFP* line was established by genetic cross in F_3_ generation. All experiments were performed under long day conditions (16 hours light/8 hours dark at 22°C). 12 μM DEX was used for TCP4 induction in all the experiments unless specified otherwise.

### Plasmid constructs and transgenic lines

The 2.16 kb upstream region of *TCP4* including 5’ untranslated region was amplified using the forward primer 5’-AATTGACCCTTTTCTATCATGC-3’ and the reverse primer 5’-TGGTAGAGCATATTCGTCGAGA-3’, *pfu* DNA polymerase and Col-0 genomic DNA and cloned into *pGEMT-Easy* vector by TA cloning (Promega; *pGEMT-Easy-ProTCP4*) [27] and then moved into *pCAMBIA 1304* using NcoI and SacI digestion to generate *ProTCP4*:*GUS* construct.

The *TCP4* cDNA fragment of *pBSKS-TCP4*:*GR* [27] was digested with the SalI and BamHI and moved into *pCAMBIA-1391Xa* to generate *TCP4*:*GUS* cassette. The NotI released 2.16 kb *ProTCP4* fragment of *pGEMT-Easy-ProTCP4*, described above, was end filled with Klenow DNA polymerase and cloned upstream of the *TCP4*:*GUS* cassette by blunt end ligation to generate *ProTCP4*:*TCP4*:*GUS* construct. The transgenic lines were generated in Col-0 background by Agrobacterium tumefaciens-mediated floral dip method [69].

### Measurement of leaf size, epidermal cell size and cell number

For the measurement of leaf size the crinkled leaves were flattened and photographed using a Canon PowerShot S110 camera. Leaf area measurement was made in the photographs using Image J software (*rsbweb*.*nih*.*gov/ij/)*. The first leaf from 29-day old plants were cleared with 70% alcohol and processed in choral hydrate:water:glycerol (8:2:1) solution and lactic acid for one to three weeks. In another method, leaves were collected in 70% ethanol followed by shifting them to ethanol:glacial acetic acid (7:1) and kept overnight (12–14 h) at room temperature. After clearing, the leaf tissues were treated with 1 M KOH for maximum 30 min in constant rotating followed by two times washing with double-distilled water. Image of abaxial epidermal cells were taken using differential interference contrast microscope (Olympus, USA) and analyzed with Image J software. For cell size analysis, 400–750 cells area was measured from different fields of 4–7 leaves and averaged to obtain the final cell size. The total number of cells per leaf was calculated by using the average cell size and leaf area.

### Kinematic analysis

The kinematic analysis of Col-0 and mock and DEX-grown *jaw-D;GR* leaf development was performed as described earlier [5,9,41,70]. Initially seedlings were grown on Murashige Skoog (MS) medium up to 10 days and then the plants were transplanted to soil. For DEX induction, the MS medium was supplemented with 12 μM DEX or ethanol control. For continuous DEX induction, seedlings were transplanted to DEX (12 μM)- or ethanol (0.04%)-treated soil and sprayed with DEX or ethanol on alternate days until experiment was completed. One of the first leaf pair from at least 9–12 plants was used for each time point of kinematic study. The leaf area, cell number and cell size were analyzed as described above. The stomatal index was analyzed as described earlier [5,41].

### Gene expression and GUS reporter analyses

Total RNA samples were isolated by Trizol (Sigma, USA) method after treatment with ethanol solvent (Mock) or 12 μM dexamethasone. To check the relative expression of respective genes, quantitative RT-PCR was performed using SYBER Green qPCR kit (SensiFAST SYBR Lo-ROX Kit, Bioline, USA) according to the manufacturer’s instructions. Results were analyzed using ABI Prism 7900HT SDS software (Applied Biosystems, USA) and ΔΔCT values were determined after normalization with internal control. Intensity ratios were calculated using the formula 2^-ΔΔCT^. Detailed experimental protocols for dexamethasone induction and primer sequences for RT-qPCR were described earlier [27,70]. GUS assay, EMSA and FAIRE experiments were performed according to the earlier protocols [9,27]. The list of primers used in this study is provided in S2 Table.

### Flow cytometry

1^st^ pair of leaves from 8-day old seedlings of indicated genotypes were collected after removing hypocotyl and cotyledons. Ploidy levels were measured using the protocol provided earlier [71,72]. In brief, leaves were placed in ice-cold Galbraith’s buffer (45 mM MgCl_2_, 30 mM sodium citrate, 20 mM 4-morpholinepropane sulfonate, and 1% Triton X-100, pH 7.0) and finely chopped using sharp razor blade followed by homogenizing in the buffer. The homogenate was filtered through Miracloth (22–25 μm) and treated with 10 μl/ml RNaseA (1mg/ml stock) for 20–30 min followed by nuclear DNA staining with 50 μl/ml of propidium iodide (1 mg/ml stock) and kept in dark at 4°C for 30 min. Samples were run through BD FACS verse (USA) flow cytometer and analyzed using BD FACS unit software. It was considered that the left-most peak corresponds to 2C [71,72]

### Chromatin immunoprecipitation (ChIP) assay

ChIP protocol was followed as mentioned earlier [39]. In brief, approximately 1.5–2 gm of fresh 10-day old *Pro35S*:*TCP4*:*3F6H* seedlings were harvested and crushed into fine powder in liquid nitrogen. The powder was homogenized in the nuclei extraction buffer 1 (0.4 M sucrose, 10 mM Tris-HCl pH 8.0, 10 mM MgCl_2_, 5 mM β-mercaptoethanol, 0.1 mM PMSF, 1 mM Na_3_VO_4_, 1 mM NaF, and Complete protease inhibitor cocktail tablets [Roche]) and proceeded for cross-linking. 37% formaldehyde was used for crosslinking by gentle rotating in 4°C for 10 min and 2 M glycine was added to stop the reaction. After quenching the cross-linked samples were filtered through two layers of Miracloth and centrifuged for 10 min at 10 k rpm at 4°C. The pellets containing the nuclei were resuspended and washed twice with extraction buffer 2 (0.25 M sucrose, 10 mM Tris-HCl pH 8.0, 10 mM MgCl_2_, 1% (w/v) Triton X-100, 5 mM β-mercaptoethanol, 0.1 mM PMSF, 1 mM Na_3_VO_4_, 1 mM NaF, and Complete protease inhibitor cocktail tablets) and transferred to ice-cold 1.5mL Eppendorf tubes followed by centrifugation at maximum rotor speed for 10 min at 4°C to isolate the nuclei. The isolated nuclei were lysed using Nuclei lysis buffer (50 mM Tris-HCl pH 8.0, 10 mM EDTA, 1% SDS, 1 mM PMSF, and Complete protease inhibitor cocktail tablets) and proceeded for sonication to shear the chromatin (~ 500 bp to 1 kb size fragments) using Bioruptor (Condition; 30 sec ON 45 sec OFF, 45 cycles). The sheared chromatin were diluted in ChIP dilution buffer (16.7 mM Tris-HCl pH 8.0, 167 mM NaCl, 1.1% Triton X-100, 1.2 mM EDTA, 0.1 mM PMSF, 1 mM Na_3_VO_4_, 1 mM NaF, and Complete protease inhibitor cocktail tablets) to make the volume up to 1.5 mL and centrifuged at maximum speed for 5 min at 4°C. From the collected chromatin solution, 30 μL (2%) was kept as input for the assay and rest of the solution was divided into two parts from which one was immunoprecipited using anti-FLAG antibody (Sigma; F1804; 1 mg/mL) and the other using Anti-IgG antibody (Sigma; A9044). Magnetic Dynabeads Protein G (Life technologies) were used for Immunoprecipitation for which first the beads were pretreated with the anti-FLAG antibody (Sigma; F1804; 1 mg/mL) or anti-IgG antibody for 20 min by rotating at room temperature and diluted in ChIP dilution buffer after washing twice with 1X PBST. The antibody-bound beads were incubated with chromatin solution (26 μL Dynabeads and 3 μL antibody per 600 μL of chromatin solution) for 20 min in ice followed by 2 h rotating at 4°C. After immunoprecipitation, the immunocomplex was washed using low salt buffer (20 mM Tris-HCl pH 8.0, 150 mM NaCl, 0.1% SDS, 1% Triton X-100, 2 mM EDTA) and high salt buffer (20 mM Tris-HCl pH 8.0, 500 mM NaCl, 0.1% SDS, 1% Triton X-100, 2 mM EDTA) and TE buffer (10 mM Tris-HCl pH 8.0, 1 mM EDTA). To elute the immunocomplex from the beads, 50 μL of nuclei lysis buffer was added and incubated for 20 min at 65°C and the elution was repeated twice. Eluted immunocomplex along with the input samples were reverse cross-linked by adding 6 μL of 5 M NaCl and incubating at 65°C overnight. Proteinase K and RNase A was treated to digest all proteins and RNA, respectively, and finally DNA were cleaned up by phenol:chloroform:isoamyl alcohol (15:24:1) followed by precipitation using sodium acetate and 100% ethanol. The final extracted DNA was dissolved in 50 μL of nuclease-free water and proceeded for qPCR using 0.8 μL DNA as template per reaction.

To quantify the relative enrichment on the respective gene element, qPCR was performed using 0.8 μL of immunoprecipited DNA and SYBER Green qPCR kit (SensiFAST SYBR Lo-ROX Kit, Bioline, USA) in a 10 μL reaction volume according to manufacturer’s protocol. Relative enrichment was calculated using the formula 0.02 X 2^(Ct input- Ct IP)^ X 100 and normalized to IgG control. The final fold enrichment was plotted in relative to Col-0 value.

## Supporting information

S1 Fig Effects of various gain-of-function forms of TCP4 on leaf area, cell number and cell size. (A) Mature first leaves from 29-day old *Pro35S*:*mTCP4*:*GR #2* plants grown in the absence (Mock) or presence (DEX) of 12 μM dexamethasone. (B) Level of *TCP4* transcript (relative to *PP2A*) in 9-day old seedlings of indicated genotypes analyzed by RT-qPCR. Averages of three biological replicates are shown. (C) to (E) Averages of leaf area (C), cell number (D) and abaxial pavement cell size (E) of leaves shown in (A). Sample number, 20. (F) to (K) Mature first leaves (F) collected from 29-day old plants, their area (G), total cell number (H), relative cell number reduction to Col-0 (I), abaxial pavement cell size (J) and relative cell area to Col-0 (K). Sample number, 8 to 12 leaves. Error bars indicate SD. * indicates p <0.05. Unpaired Student’s *t*-test was used. For (E) and (J), 120–140 cells per leaf were measured and averages of 3–4 leaves are shown. Scale bars in (A, F), 2.5 mm.
(TIF)

S2 Fig MiR319-targeted *TCP* genes are essential and sufficient for cell differentiation. (A) Outline of epidermal cells on the abaxial surface of mature first leaf. (B) Area of pavement cells in mature first leaves from DEX-treated Col-0;*ProTCP4*:*mTCP4*:*GR* and *jaw-D*;*ProTCP4*:*mTCP4*:*GR* plants relative to the mock-treated values. (C) Frequency distribution of abaxial pavement cell size in the first leaf shown in (A). Total 120–150 cells per leaf were measured and averages from 5 leaves are shown. (D) Frequency distribution of pavement cell size on the abaxial surface of mature first leaf from Col-0;*ProTCP4*:*mTCP4*:*GR* plants grown in the absence (Mock) or presence (DEX) of dexamethasone. Error bars indicate SD. * indicates p < 0.05. Unpaired Student’s *t*-test was performed. Scale bars, 100 μm (A).
(TIF)

S3 Fig Kinematic growth analysis of leaves with altered *TCP4* activity. (A) Average width of the first leaf pair of Col-0 and *jaw-D;ProTCP4*:*mTCP4*:*GR* plants grown in the absence (Mock) or presence (DEX) of 12 μM dexamethasone. (B) Schematic of a leaf (left) to highlight the region on the abaxial surface (yellow square) used for cell size analysis and morphology of epidermal cells on the abaxial surface of the first leaf pair of Col-0 in the corresponding regions at two different growth stages (right). (C) to (E) Proportion of smaller (<1500 μm^2^) and large (>1500 μm^2^) cells on the abaxial surface of first leaf at different days after stratification in Col-0 (C) plants and *jaw-D;ProTCP4*:*mTCP4*:*GR* (*jaw-D;GR*) plants grown in the absence (Mock, D) or presence (DEX, E) of dexamethasone. N, 10–13 leaves. For each time point, total 30–40 cells per leaf at specified region shown in (B) were measured and averages from 5–7 leaves shown. Error bars indicate SD.
(TIF)

S4 Fig Schematic representation of the treatment regime of dexamethasone. (A) and (B) TCP4 function was induced in the *jaw-D;ProTCP4*:*mTCP4*:*GR* plants by shifting the seedlings from Mock→DEX (A) or DEX→Mock (B) at indicated days after stratification (DAS). All the leaf parameters shown in Fig 3 and Fig 4 were analyzed in the mature first leaves at 29 DAS.
(TIF)

S5 Fig Kinematic growth analysis of leaves expressing miR319-resistant/ susceptible TCP4. (A) and (B) Average area (A) of the first leaf from *jaw-D;ProTCP4*:*mTCP4*:*GR* seedlings grown in the absence of dexamethasone and then shifted to dexamethasone-containing medium at 8 or 10 days after stratification (DAS) and size of their pavement cells on the abaxial surface (B). N, 12–15 leaves. For each time point, total 30–40 cells per leaf at specified region (S2B Fig) were measured and averages from 5–7 leaves shown. The corresponding values for plants grown in continuous Mock medium (broken lines) are reproduced from Fig 2 for comparison. (C) to (F) Images of mature first leaves (C) and their average size (D) to (F) of Col-0;*ProTCP4*:*mTCP4*:*GR* (Col-0;*GR*), Col-0;*Pro35S*:*mTCP4*:*GR* (*35S;GR*) and Col-0*;Pro35S*:*TCP4*:*GR* (*35S;sGR*) plants treated with 12 μM dexamethasone for 48 hours at indicated days after stratification (DAS). Plants grown in continuous absence (M, Mock) or presence (D, DEX) of dexamethasone are shown as controls. Dotted lines indicate the values corresponding to Mock. N, 10–15 leaves. Error bars indicate SD.
(TIF)

S6 Fig Schematic representation of 24-hour dexamethasone pulse. Pulse of dexamethasone treatment was performed in the *jaw-D;ProTCP4*:*mTCP4*:*GR* plants by shifting the seedlings from Mock→DEX for 24 hours at indicated days after stratification (DAS) and then again to Mock condition. Mature first leaf size was analyzed at 29 DAS.
(TIF)

S7 Fig Commitment to differentiation in leaf pavement cells by TCP4. (A) Mature first leaves of 29-day old *Pro35S*:*mTCP4*:*GR #2* plants grown either in the total absence of dexamethasone (Mock) or in the presence of 12 μM dexamethasone (DEX) for the indicated number of days and then shifted to Mock till 29 DAS. (B) Average area (N = 10–15) of leaves shown in (A). The dotted line is drawn through the Mock value parallel to the X-axis.
(TIF)

S8 Fig Ectopic miR319 abolishes TCP4 from the transition zone. GUS reporter analysis of the first leaf pair in 4-day old seedlings grown in the absence of dexamethasone. All genotypes were analyzed in the F1 generation. Numbers indicate leaf length in mm.
(TIF)

S9 Fig Differential expression of 29 *SAUR* transcripts and 3 *HD-ZIP II* transcripts upon 2 h and 4 h of TCP4 induction in the *jaw-D;ProTCP4:mTCP4:GR* seedling as found in a previously reported microarray dataset [27]. (TIF)

S10 Fig *HAT2* FAIRE results and *HAT1* locus. (A) Quantitative PCR analysis of the *HAT2* upstream regulatory regions (R1-R3 shown in Fig 7I) by FAIRE experiment on chromatin DNA isolated from 10-day old *Pro35S*:*mTCP4*:*GR* seedlings before (Mock) or after (DEX) 12 μM dexamethasone treatment for 4 h. *YUC5* was used as a positive control [27] and R3 serves as an internal negative control. All values were normalized to *TA3*. Averages of biological triplicates are shown. Error bars indicate SD. ns, not significant, * indicates p <0.05, unpaired Student’s *t*-test was used. (B) A schematic representation of *HAT1* genomic structure. Exons are shown in gray boxes and the translation start site is shown by an arrow. Two putative TCP4 DNA-binding motifs (TGGCCC) are indicated. The four regions used for the ChIP-qPCR amplification (in C) are shown as R1-R4. (C) ChIP-qPCR analysis of *HAT1* locus (R1-R4 in B) with anti-FLAG antibody. *LOX2* and *TUB2* were used as positive and negative controls, respectively (shown in Fig 7K, since this experiment was performed together with the *HAT2* ChIP experiment). Averages of biological triplicates of qPCR analysis are shown.
(TIF)

S11 Fig Expression analysis of *TCP4*, *HAT1* and *HAT2* at various developmental stages. (A) and (B) Levels of *TCP4*, *HAT1* and *HAT2* transcripts at various developmental stages as analyzed by *Genevestigator* tool (https://genevestigator.com/gv/doc/intro_plant.jsp) (A) and estimated by RT-qPCR (B). For (B), RNA samples were isolated from seedlings (2, 4 and 6 DAS) and from first pair of leaves (8, 10 and 14 DAS). *PP2A* was used as an internal control. Error bars indicate SD.
(TIF)

S12 Fig Partial rescue of *jaw-D* phenotype by *HAT1* overexpression. (A) to (F) 30-day old first leaves (A), their average area (B), total pavement cell number (C), cell number relative to Col-0 (D), outline of abaxial pavement cells (E) and their average area (F). N, 8–12 leaves. *jaw-D;GR* and *JAB*:*GFP* indicate *jaw-D;ProTCP4*:*mTCP4*:*GR* and *Pro35S*:*JAB*:*GFP*, respectively. For (F) total 125–150 cells per leaf were measured and averages from 3–7 leaves shown. Error bars indicate SD. * indicates p <0.05. Unpaired Student’s *t*-test was used.
(TIF)

S1 Table Kinematic data shown in Fig 2C. (XLSX)

S2 Table List of primers used in this study. (XLSX)
