# Supplementary figures and images for: The CIN-TCP transcription factors promote commitment to differentiation in Arabidopsis leaf pavement cells via both auxin-dependent and independent pathways
Source: PLoS Genet. 2019 Feb 11;15(2):e1007988. doi: 10.1371/journal.pgen.1007988 (PMC6386416; doi:10.1371/journal.pgen.1007988)

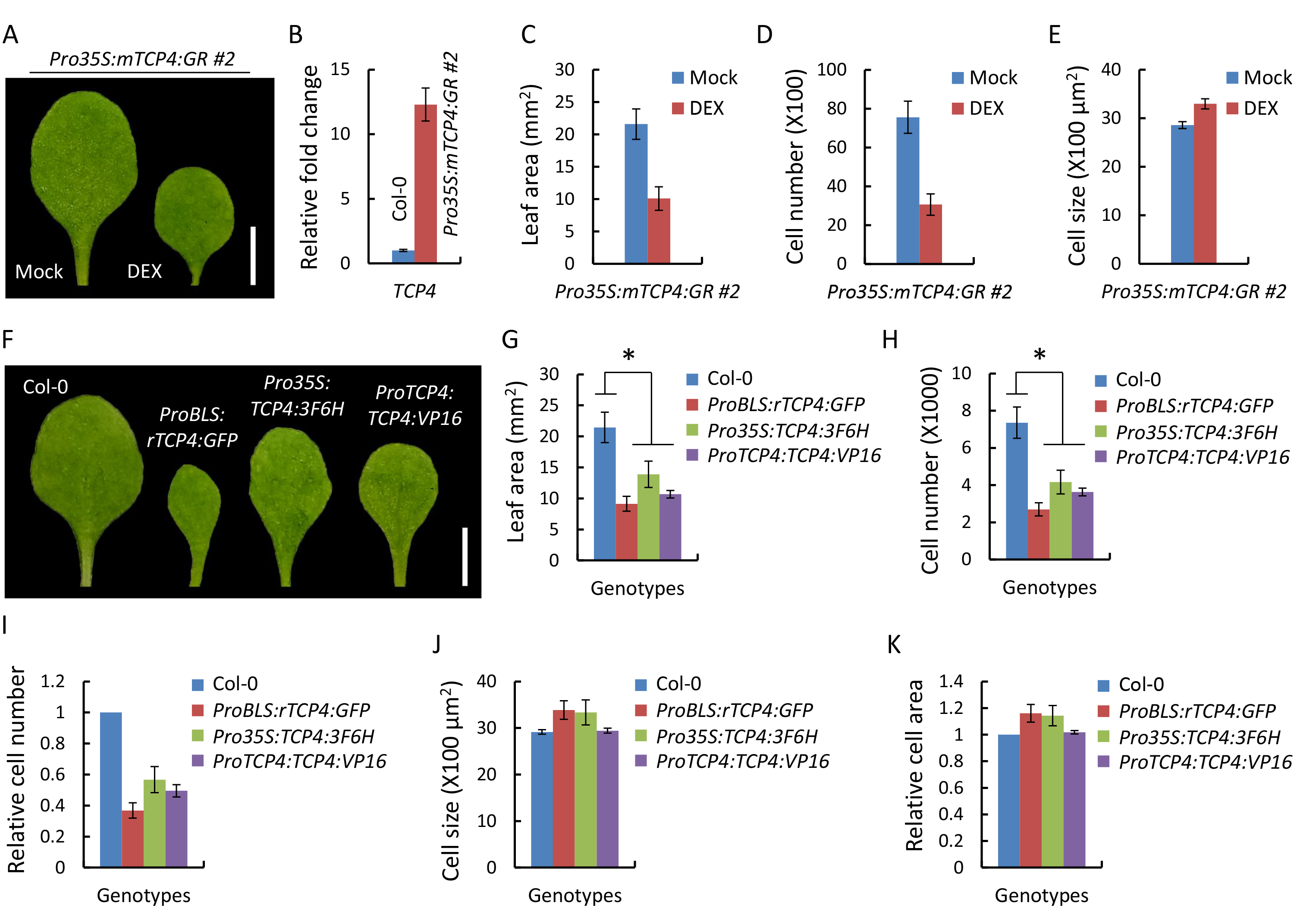

Supplement: S1 Fig — (A) Mature first leaves from 29-day old Pro35S:mTCP4:GR #2 plants grown in the absence (Mock) or presence (DEX) of 12 μM dexamethasone. (B) Level of TCP4 transcript (relative to PP2A) in 9-day old seedlings of indicated genotypes analyzed by RT-qPCR. Averages of three biological replicates are shown. (C) to (E) Averages of leaf area (C), cell number (D) and abaxial pavement cell size (E) of leaves shown in (A). Sample number, 20. (F) to (K) Mature first leaves (F) collected from 29-day old plants, their area (G), total cell number (H), relative cell number reduction to Col-0 (I), abaxial pavement cell size (J) and relative cell area to Col-0 (K). Sample number, 8 to 12 leaves. Error bars indicate SD. * indicates p <0.05. Unpaired Student’s t-test was used. For (E) and (J), 120–140 cells per leaf were measured and averages of 3–4 leaves are shown. Scale bars in (A, F), 2.5 mm. (TIF) [file pgen.1007988.s001.tif]

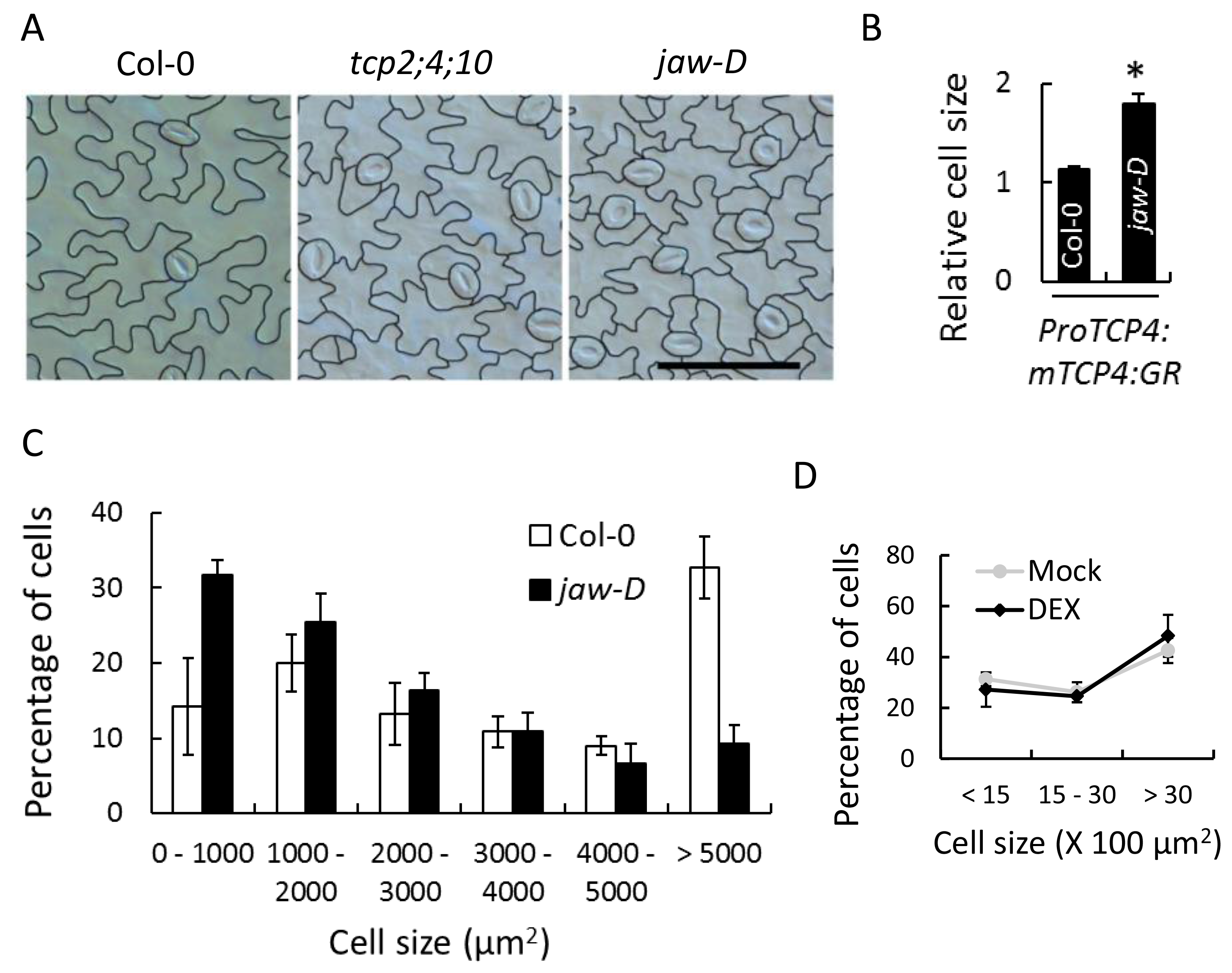

Supplement: S2 Fig — (A) Outline of epidermal cells on the abaxial surface of mature first leaf. (B) Area of pavement cells in mature first leaves from DEX-treated Col-0;ProTCP4:mTCP4:GR and jaw-D;ProTCP4:mTCP4:GR plants relative to the mock-treated values. (C) Frequency distribution of abaxial pavement cell size in the first leaf shown in (A). Total 120–150 cells per leaf were measured and averages from 5 leaves are shown. (D) Frequency distribution of pavement cell size on the abaxial surface of mature first leaf from Col-0;ProTCP4:mTCP4:GR plants grown in the absence (Mock) or presence (DEX) of dexamethasone. Error bars indicate SD. * indicates p < 0.05. Unpaired Student’s t-test was performed. Scale bars, 100 μm (A). (TIF) [file pgen.1007988.s002.tif]

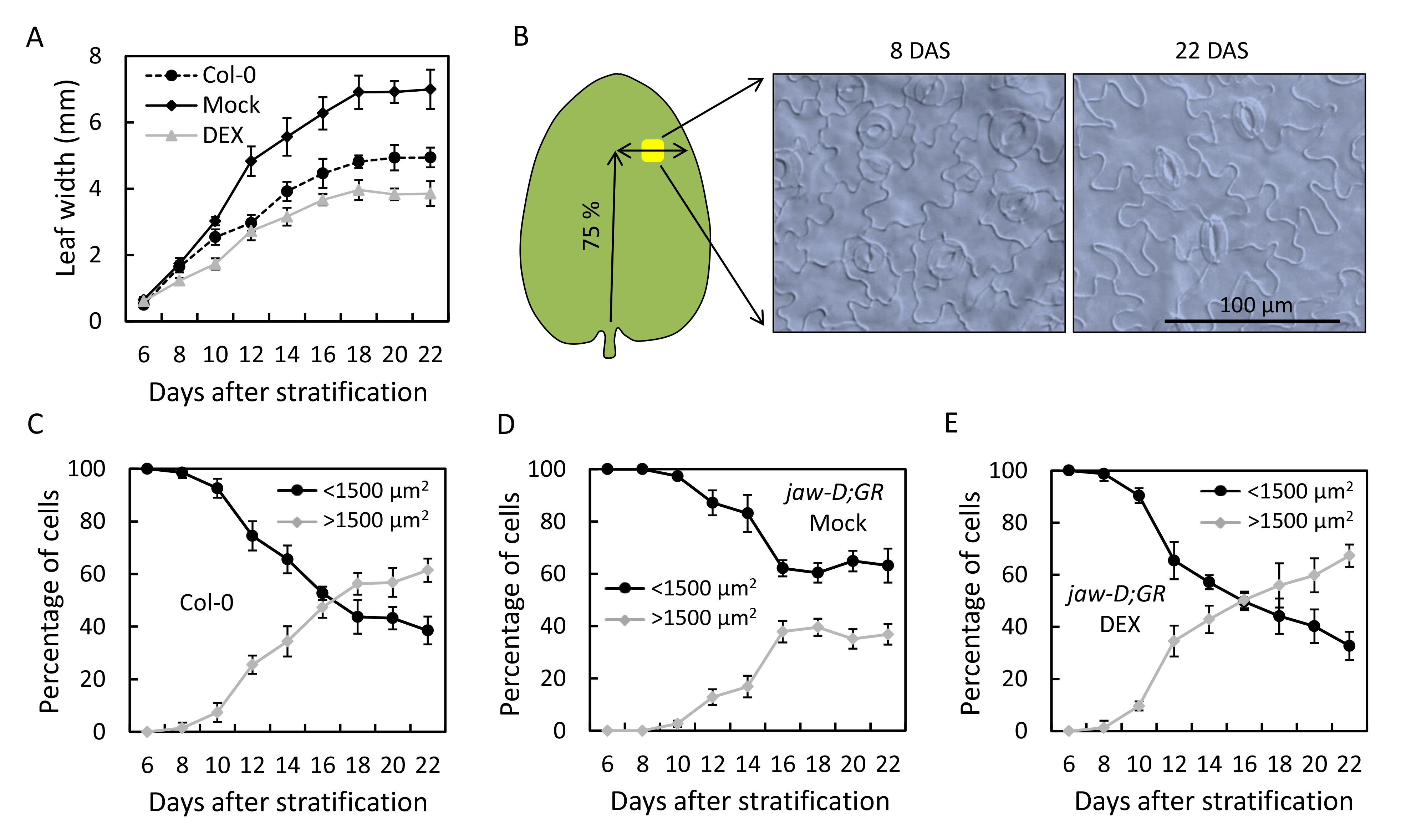

Supplement: S3 Fig — (A) Average width of the first leaf pair of Col-0 and jaw-D;ProTCP4:mTCP4:GR plants grown in the absence (Mock) or presence (DEX) of 12 μM dexamethasone. (B) Schematic of a leaf (left) to highlight the region on the abaxial surface (yellow square) used for cell size analysis and morphology of epidermal cells on the abaxial surface of the first leaf pair of Col-0 in the corresponding regions at two different growth stages (right). (C) to (E) Proportion of smaller (<1500 μm2) and large (>1500 μm2) cells on the abaxial surface of first leaf at different days after stratification in Col-0 (C) plants and jaw-D;ProTCP4:mTCP4:GR (jaw-D;GR) plants grown in the absence (Mock, D) or presence (DEX, E) of dexamethasone. N, 10–13 leaves. For each time point, total 30–40 cells per leaf at specified region shown in (B) were measured and averages from 5–7 leaves shown. Error bars indicate SD. (TIF) [file pgen.1007988.s003.tif]

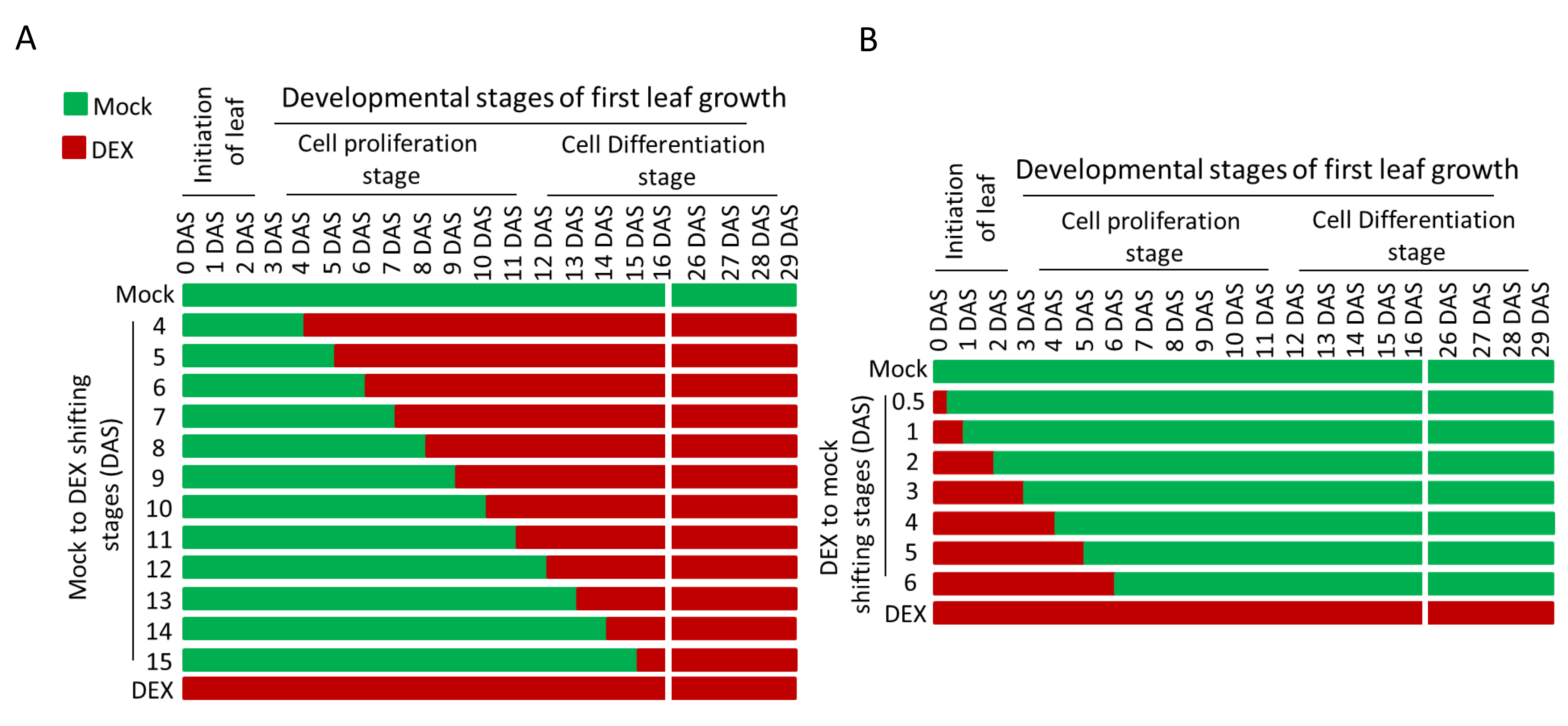

Supplement: S4 Fig — (A) and (B) TCP4 function was induced in the jaw-D;ProTCP4:mTCP4:GR plants by shifting the seedlings from Mock→DEX (A) or DEX→Mock (B) at indicated days after stratification (DAS). All the leaf parameters shown in Fig 3 and Fig 4 were analyzed in the mature first leaves at 29 DAS. (TIF) [file pgen.1007988.s004.tif]

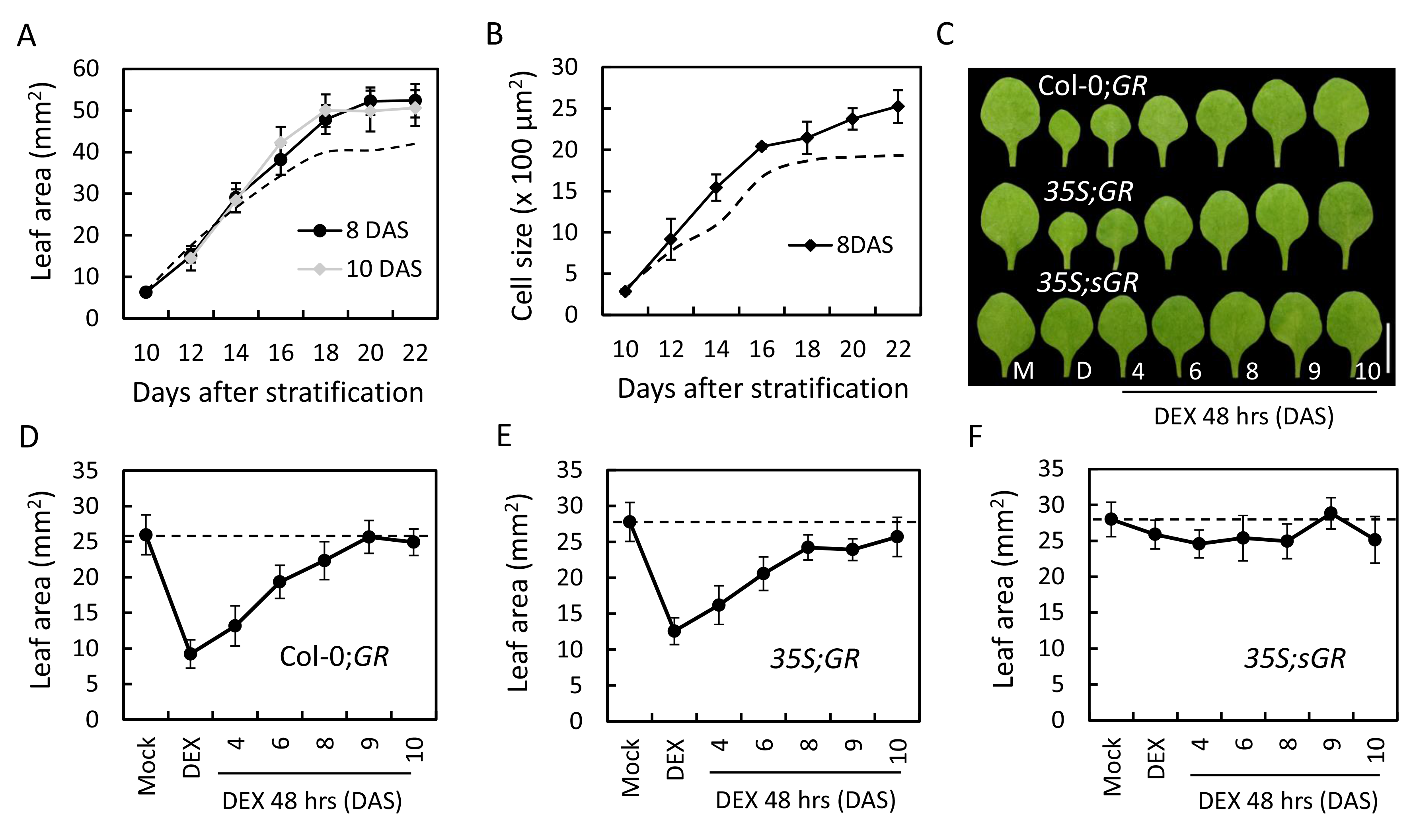

Supplement: S5 Fig — (A) and (B) Average area (A) of the first leaf from jaw-D;ProTCP4:mTCP4:GR seedlings grown in the absence of dexamethasone and then shifted to dexamethasone-containing medium at 8 or 10 days after stratification (DAS) and size of their pavement cells on the abaxial surface (B). N, 12–15 leaves. For each time point, total 30–40 cells per leaf at specified region (S2B Fig) were measured and averages from 5–7 leaves shown. The corresponding values for plants grown in continuous Mock medium (broken lines) are reproduced from Fig 2 for comparison. (C) to (F) Images of mature first leaves (C) and their average size (D) to (F) of Col-0;ProTCP4:mTCP4:GR (Col-0;GR), Col-0;Pro35S:mTCP4:GR (35S;GR) and Col-0;Pro35S:TCP4:GR (35S;sGR) plants treated with 12 μM dexamethasone for 48 hours at indicated days after stratification (DAS). Plants grown in continuous absence (M, Mock) or presence (D, DEX) of dexamethasone are shown as controls. Dotted lines indicate the values corresponding to Mock. N, 10–15 leaves. Error bars indicate SD. (TIF) [file pgen.1007988.s005.tif]

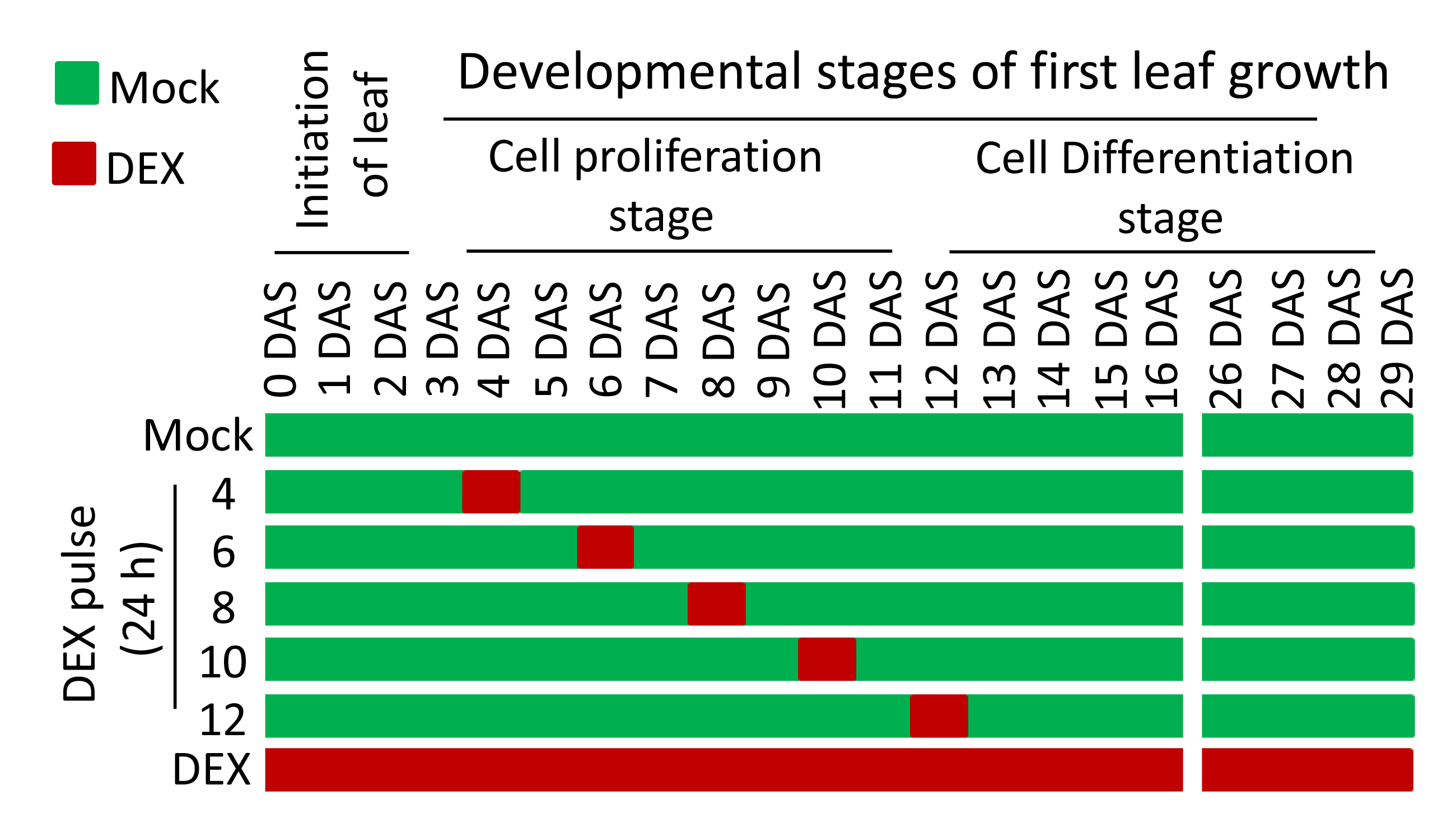

Supplement: S6 Fig — Pulse of dexamethasone treatment was performed in the jaw-D;ProTCP4:mTCP4:GR plants by shifting the seedlings from Mock→DEX for 24 hours at indicated days after stratification (DAS) and then again to Mock condition. Mature first leaf size was analyzed at 29 DAS. (TIF) [file pgen.1007988.s006.tif]

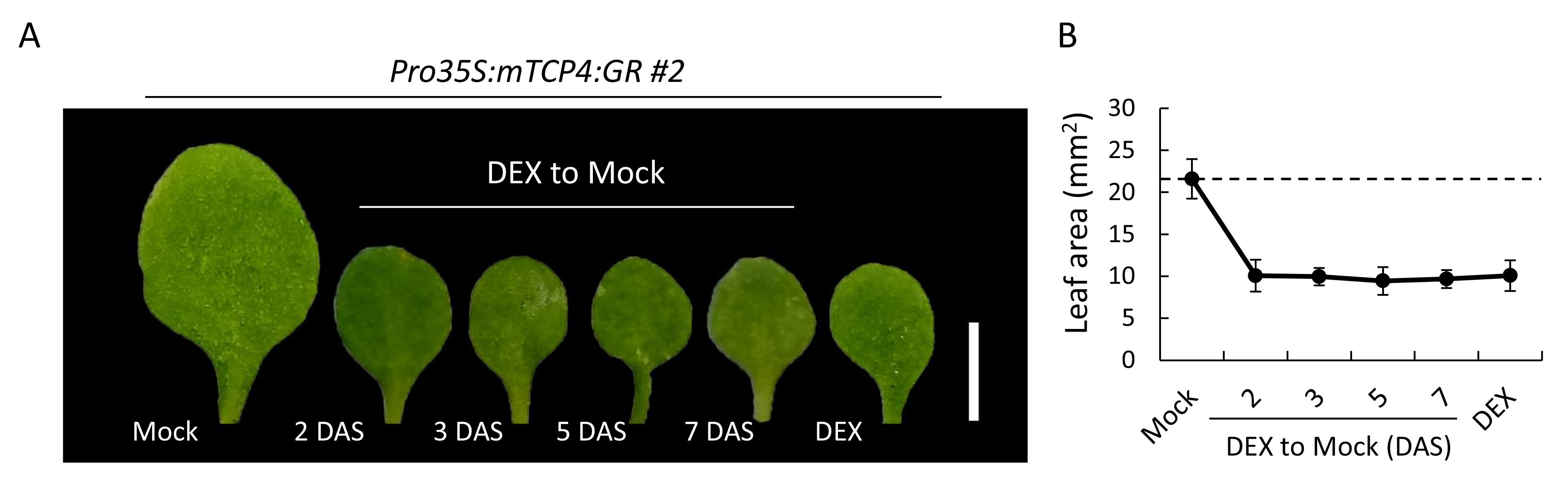

Supplement: S7 Fig — (A) Mature first leaves of 29-day old Pro35S:mTCP4:GR #2 plants grown either in the total absence of dexamethasone (Mock) or in the presence of 12 μM dexamethasone (DEX) for the indicated number of days and then shifted to Mock till 29 DAS. (B) Average area (N = 10–15) of leaves shown in (A). The dotted line is drawn through the Mock value parallel to the X-axis. (TIF) [file pgen.1007988.s007.tif]

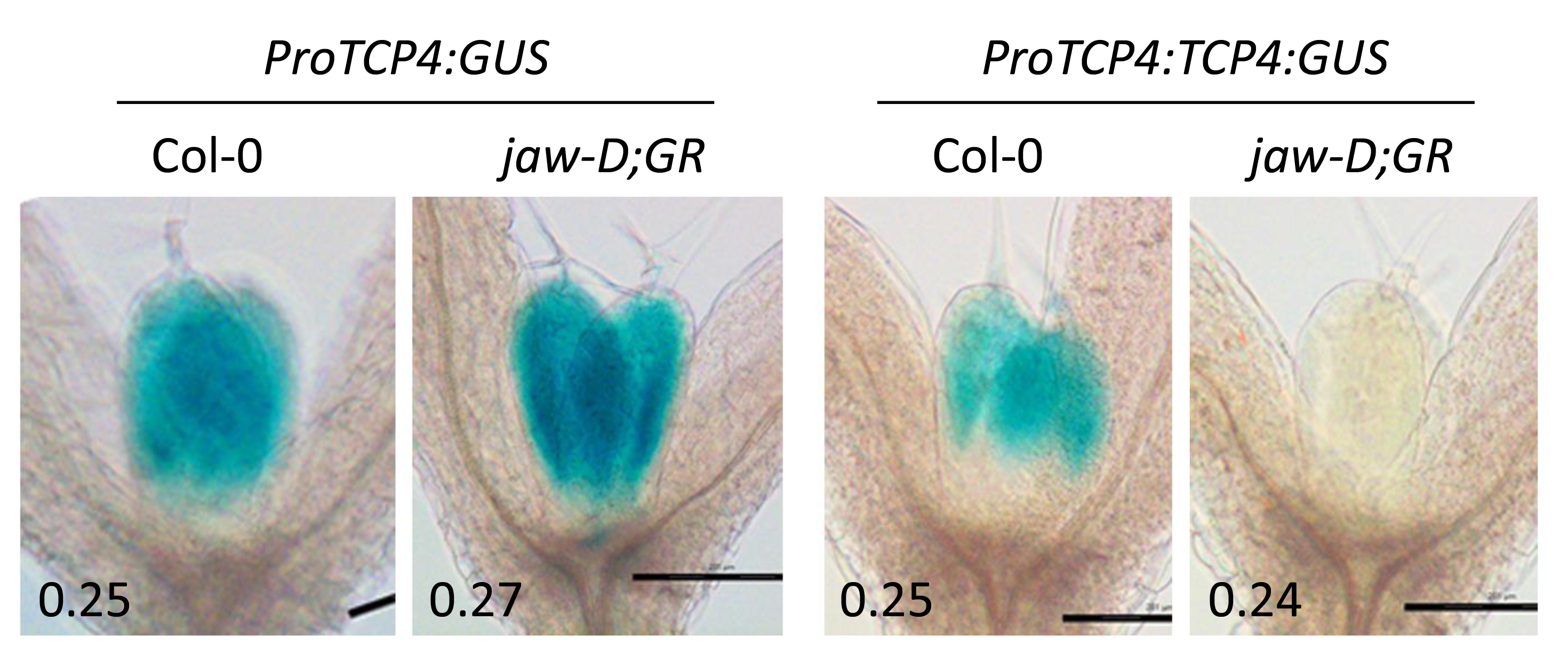

Supplement: S8 Fig — GUS reporter analysis of the first leaf pair in 4-day old seedlings grown in the absence of dexamethasone. All genotypes were analyzed in the F1 generation. Numbers indicate leaf length in mm. (TIF) [file pgen.1007988.s008.tif]

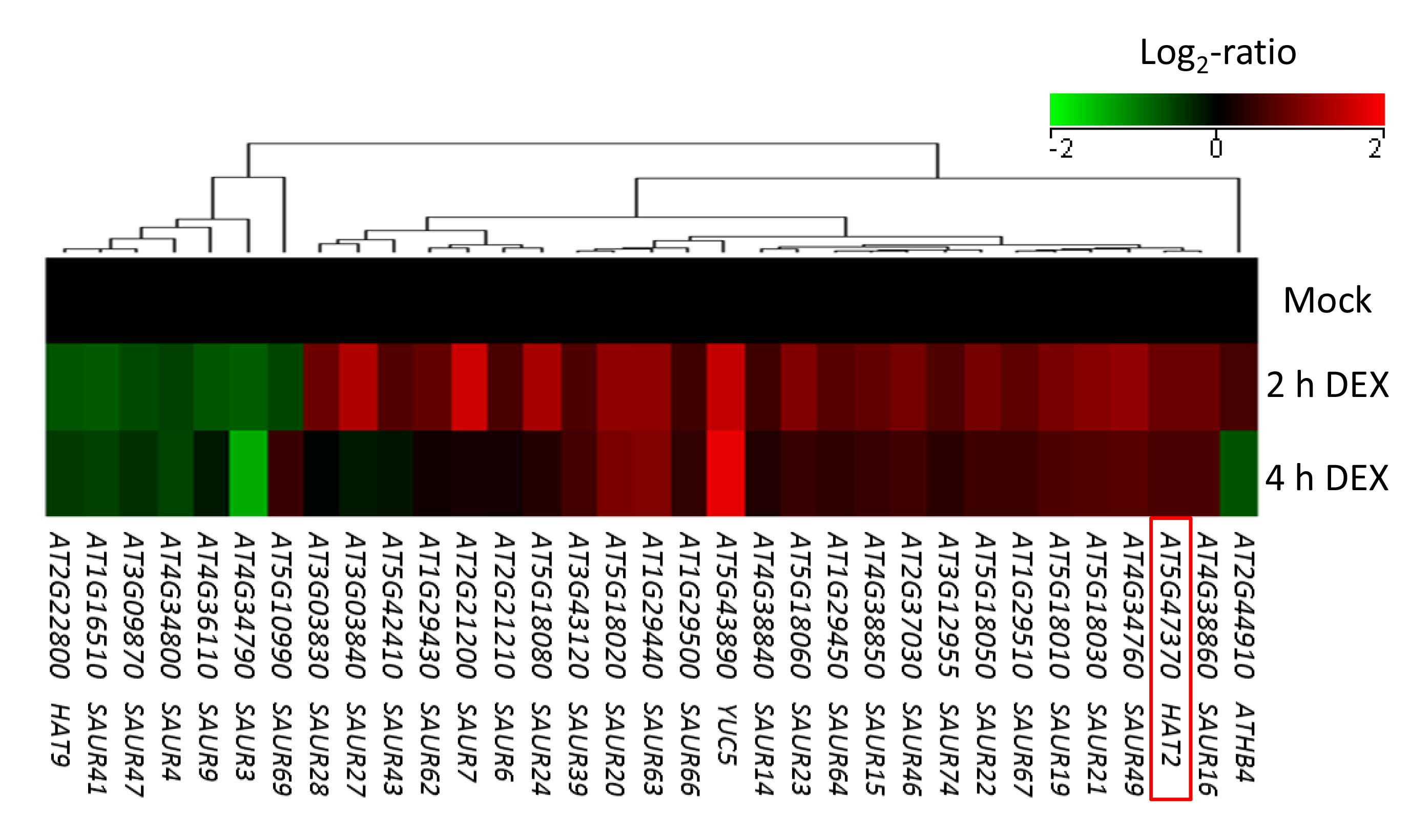

Supplement: S9 Fig — (TIF) [file pgen.1007988.s009.tif]

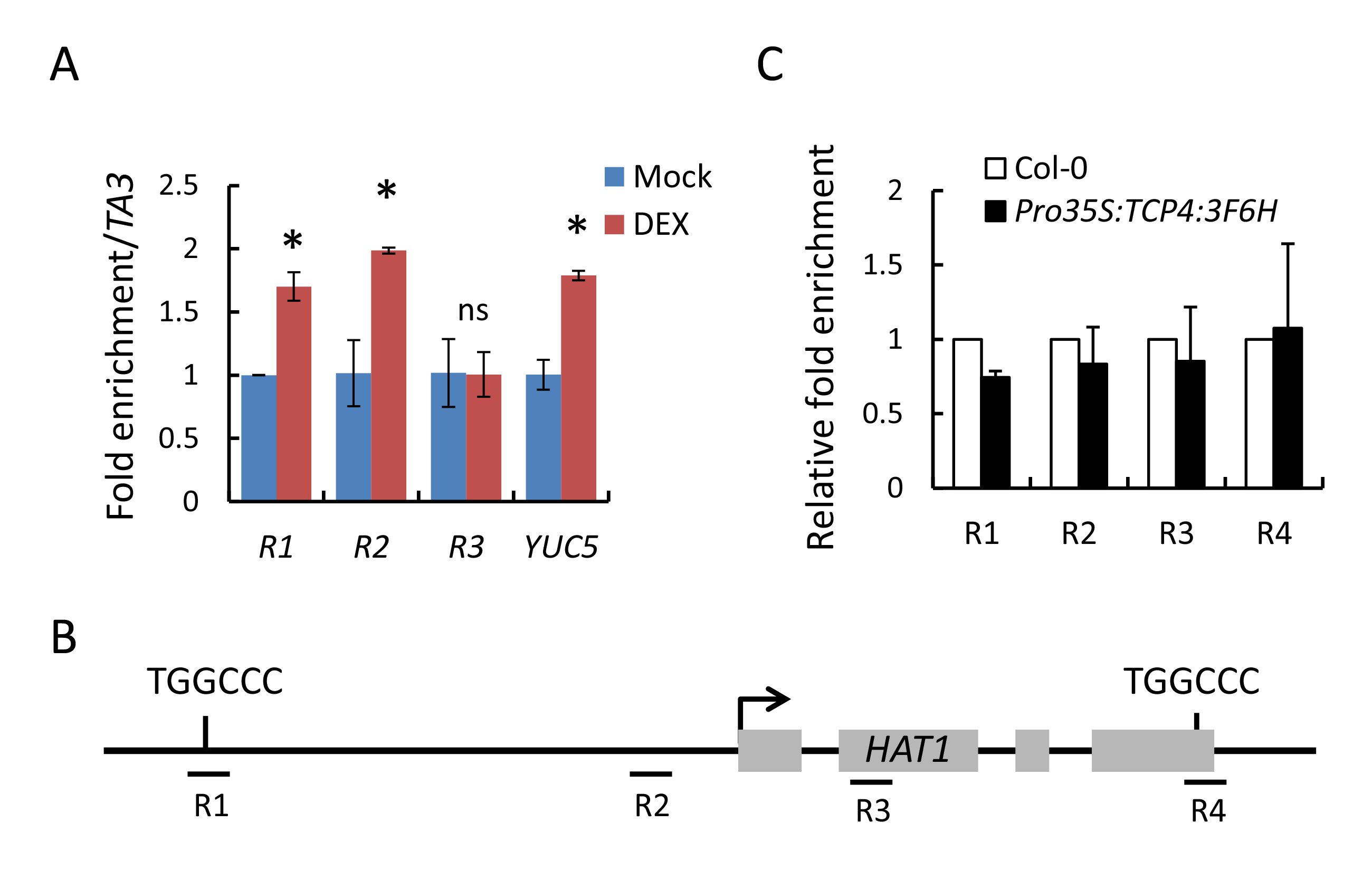

Supplement: S10 Fig — (A) Quantitative PCR analysis of the HAT2 upstream regulatory regions (R1-R3 shown in Fig 7I) by FAIRE experiment on chromatin DNA isolated from 10-day old Pro35S:mTCP4:GR seedlings before (Mock) or after (DEX) 12 μM dexamethasone treatment for 4 h. YUC5 was used as a positive control [27] and R3 serves as an internal negative control. All values were normalized to TA3. Averages of biological triplicates are shown. Error bars indicate SD. ns, not significant, * indicates p <0.05, unpaired Student’s t-test was used. (B) A schematic representation of HAT1 genomic structure. Exons are shown in gray boxes and the translation start site is shown by an arrow. Two putative TCP4 DNA-binding motifs (TGGCCC) are indicated. The four regions used for the ChIP-qPCR amplification (in C) are shown as R1-R4. (C) ChIP-qPCR analysis of HAT1 locus (R1-R4 in B) with anti-FLAG antibody. LOX2 and TUB2 were used as positive and negative controls, respectively (shown in Fig 7K, since this experiment was performed together with the HAT2 ChIP experiment). Averages of biological triplicates of qPCR analysis are shown. (TIF) [file pgen.1007988.s010.tif]

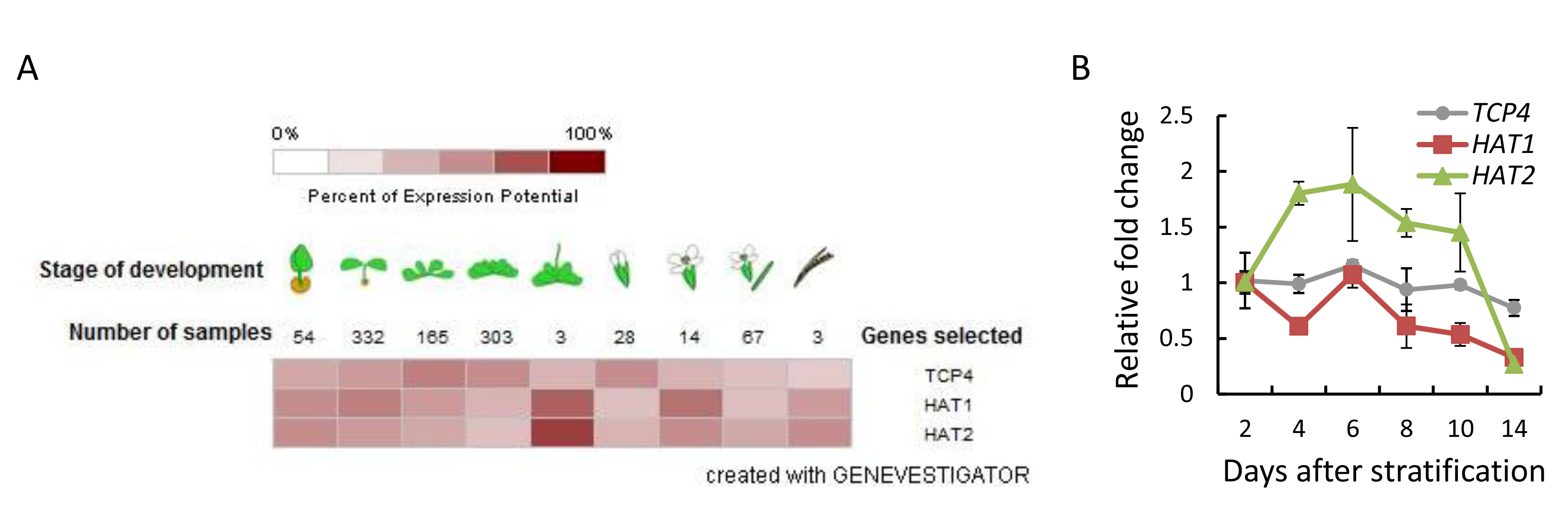

Supplement: S11 Fig — (A) and (B) Levels of TCP4, HAT1 and HAT2 transcripts at various developmental stages as analyzed by Genevestigator tool (https://genevestigator.com/gv/doc/intro_plant.jsp) (A) and estimated by RT-qPCR (B). For (B), RNA samples were isolated from seedlings (2, 4 and 6 DAS) and from first pair of leaves (8, 10 and 14 DAS). PP2A was used as an internal control. Error bars indicate SD. (TIF) [file pgen.1007988.s011.tif]

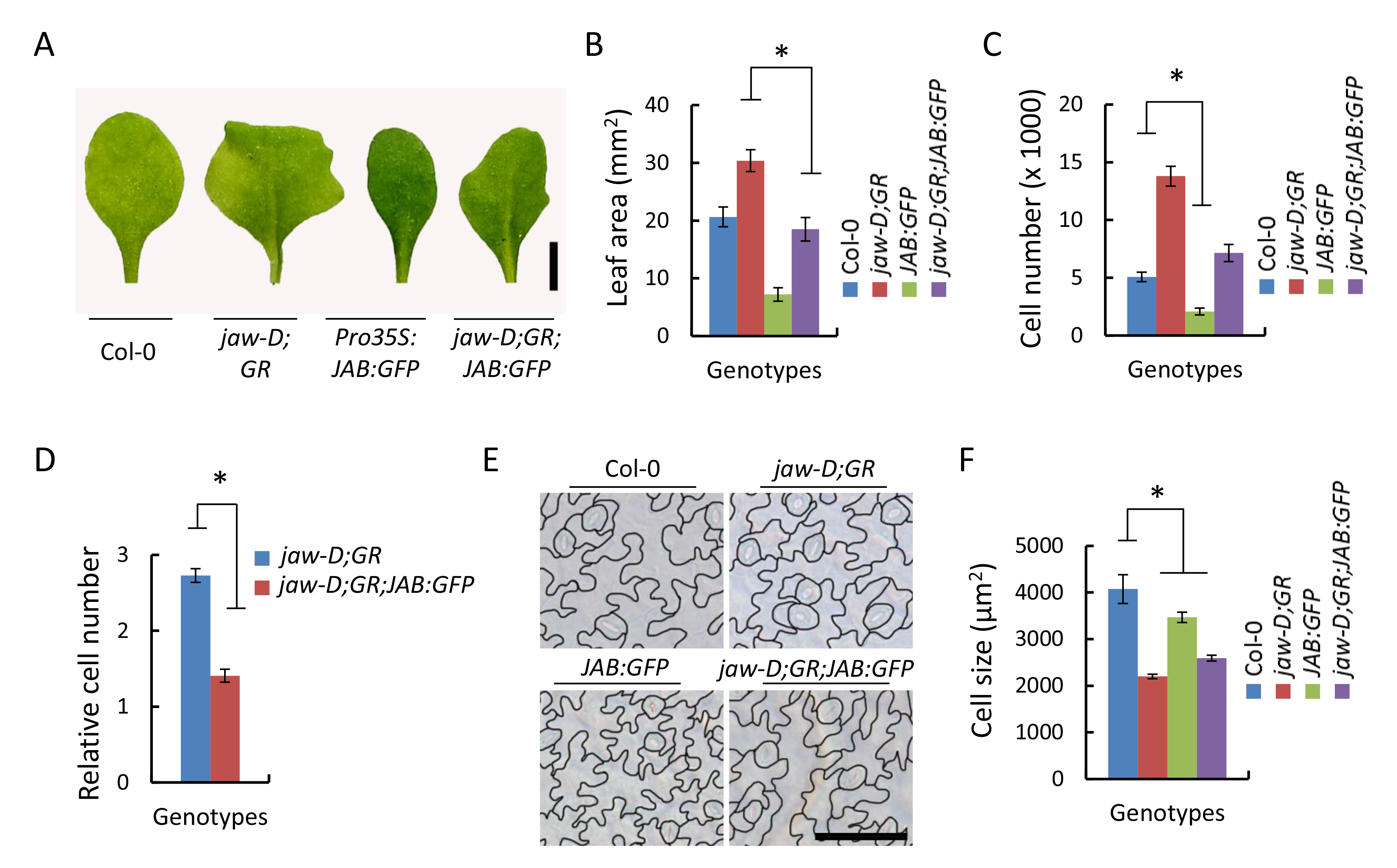

Supplement: S12 Fig — (A) to (F) 30-day old first leaves (A), their average area (B), total pavement cell number (C), cell number relative to Col-0 (D), outline of abaxial pavement cells (E) and their average area (F). N, 8–12 leaves. jaw-D;GR and JAB:GFP indicate jaw-D;ProTCP4:mTCP4:GR and Pro35S:JAB:GFP, respectively. For (F) total 125–150 cells per leaf were measured and averages from 3–7 leaves shown. Error bars indicate SD. * indicates p <0.05. Unpaired Student’s t-test was used. (TIF) [file pgen.1007988.s012.tif]
